# Videomicroscopy reveals individual response of MCF7 cells to X-ray irradiation

**DOI:** 10.1371/journal.pone.0345480

**Published:** 2026-04-15

**Authors:** Joséphine Courouble, Stéphane Plaszczynski, Olivier Seksek

**Affiliations:** 1 Université Paris-Saclay, CNRS/IN2P3, IJCLab, Orsay, France; 2 Université de Paris Cité, IJCLab, Orsay, France; Alfaisal University, College of Medicine, SAUDI ARABIA

## Abstract

Understanding the heterogeneity of cellular responses to irradiation is a key factor in cancer research to improve radiotherapy efficacy. To this aim, time-lapse 2D videomicroscopy was used to track *in vitro* more than 6 300 individual MCF7 breast cancer cells exposed to single X-ray irradiation, with doses ranging from 0 Gy to 5 Gy. Cell tracking and lineage reconstruction were performed using a dedicated algorithm: Cell Lineage Tracking (CLT). The CLT algorithm achieves high robustness and reliability across irradiation conditions, with more than 95% correct lineage tree assignment and over 80% agreement in reconstructed cell cycle duration. It allowed classification and study of the properties and fates of different cell categories (proliferative, transiently arrested and long-term arrested) and revealed distinct behavior, notably in individual cell surface area and diffusion properties. Cell surface area increased progressively across cell categories, from approximately 5 000 $\mu {\text{m}}^{2}$ in proliferative cells to about 50 000 $\mu {\text{m}}^{2}$ in long-term arrested cells, with transiently arrested cells showing intermediate values and their daughter cells approaching those of proliferative cells. Cell surface also increased with higher doses within each cell category. In contrast, diffusion decreased across cell categories, from around $0.4\;\mu {\text{m}}^{2}/\text{h }$ in proliferative cells to around $0.02\;\mu {\text{m}}^{2}/\text{h }$ in long-term arrested cells, with transiently arrested cells showing intermediate diffusion and their daughter cells approaching the dynamic behavior of proliferative cells. Within each cell category, diffusion did not show a clear dependence on dose. This novel single-cell and lineage-level framework highlights the importance of dynamic analyses in uncovering the complexity and heterogeneity of radiation responses.

## Introduction

Cancer remains one of the leading causes of morbidity and mortality worldwide, characterized by uncontrolled cell proliferation and the ability to evade mechanisms that normally restrict growth [1]. Despite major advances in therapeutic strategies (including surgery, chemotherapy, targeted therapies and radiotherapy) the complexity and heterogeneity of tumor cell responses continue to represent major challenges in cancer management [2]. Understanding how cancer cells adapt to stress conditions, and in particular to irradiation, is therefore essential for improving treatment outcomes.

Ionizing radiation is widely used in medicine, either alone or in combination with other treatments [3]. Its primary mechanism of action involves the induction of DNA damage, particularly double-strand breaks, which can lead to cell cycle arrest, senescence, or apoptosis [4]. However, the response to irradiation is far from uniform: cells within the same population can exhibit divergent fates, ranging from successful repair and continued proliferation to transient arrest or permanent growth cessation. This heterogeneity underlies treatment resistance and recurrence, making it crucial to better characterize the diversity of radiation-induced cellular responses [5,6].

Traditionally, the impact of irradiation has been assessed *in vitro* through molecular and biochemical markers such as γH2AX foci for DNA damage [7,8]. More recently, high-content imaging systems such as IncuCyte^®^ have enabled time-resolved monitoring of cell density in response to treatment [9,10]. While informative, these approaches provide population-level measurements that may mask individual cell behaviors and fail to capture dynamic transitions between phenotypic states.

Time-lapse videomicroscopy offers a powerful complementary approach by enabling direct observation of cell morphology and behaviour over extended periods [11]. Coupled with quantitative image analysis, it allows dynamic tracking of individual cells and their progeny, thereby revealing fate decisions that remain inaccessible to static endpoint assays. This methodology is particularly well-suited for studying responses to irradiation, as it can capture both early transient phenomena (e.g., cell cycle arrest and recovery) and long-term outcomes as sustained proliferation arrest [12,13].

However, segmentation and tracking of cells in time-lapse microscopy images is challenging. Each dataset presents specific characteristics: distinct cell lines, imaging modalities (e.g., brightfield, nuclear fluorescence, or cytoplasmic fluorescence), cell density, acquisition durations, and temporal resolutions. This complexity results in heterogeneous image properties and analysis requirements [14,15]. Moreover, the study objectives vary widely, making different experiments sensitive to different types of tracking errors. Investigations of irradiation effects, in particular, require highly accurate detection of cell division events while maintaining sufficient flexibility to accommodate evolving cellular phenotypes.

The segmentation step is the most critical component of a tracking pipeline, where the suite Cellpose [16,17] is today the state-of-the-art approach despite the diversity of available tools [18,19]. Current algorithms that attempt to perform segmentation and tracking simultaneously do not yet provide competitive performance [20,21]. Recent reviews consistently emphasize that no universal solution exists; each experimental setup demands its own optimized cell-tracking strategy [22]. In the rare biological videomicroscopy studies focusing on cell division tracking, tailored approaches are typically employed, often relying on semi-manual tracking. This process requires extensive manual intervention and can only be performed at a limited statistical sample [12].

In this study, for the first time, videomicroscopy and cell tracking are applied to characterize the heterogeneous responses of MCF7 breast cancer cells exposed to different radiation doses, over a large sample of more than 6 300 cells. To this aim, a novel algorithm CellLineageTrack (CLT) was developped. By combining single-cell lineage reconstruction with measurements of cell cycle duration, surface area dynamics, and motility parameters, distinct cellular fates are identified and classified, including proliferative, transiently arrested, and long-term arrested phenotypes. Importantly, a novel framework is introduced, distinguishing primary transiently arrested cells and their repaired progeny, revealing aspects of the dynamics of recovery and adaptation after irradiation. This approach provides a complex view of radiation responses of individual cells and highlights the importance of single-cell and lineage-level analyses in understanding treatment-induced heterogeneity.

To facilitate reading and explanation of the process, population-level results are first examined, serving as the basis for defining the three categories (proliferative, transiently arrested, and long-term arrested cells) based on lineage tree analysis. Each category is then described individually, before being compared in the discussion.

## Materials and methods

### Cell culture

The human breast cancer cell line MCF-7 (stably expressing Green Fluorescent Protein; Cell Biolabs, Inc., San Diego, USA; catalog no. AKR-211) was used for the experiments. Cells are grown in culture flasks (ClearLine) in DMEM medium, supplemented with 1 mM GlutaMAX, 1 mM sodium pyruvate, 10% fetal bovine serum, and 1% penicillin-streptomycin solution (10 000 units) (Gibco^TM^, Thermo Fisher Scientific, France). Cell cultures were maintained in an BINDER incubator at 37°C in a 5% CO_2_ and 95% humidity. When cells reach approximately 75% confluence, they are trypsinized using 0.05% trypsin-EDTA (Gibco^TM^, Thermo Fisher Scientific, France) and subsequently reseeded into culture flasks.

### Radiation treatment and imaging

One day prior to irradiation, cells are seeded into T12.5 flasks containing 5 mL of complete culture medium and incubated overnight. On the following morning, irradiation is performed using the Xrad 320 Dx irradiator (PXi, Madison, CT, USA) of the RadeXp platform (Institut Curie, Orsay, France). Cells are exposed to doses of 0, 1, 2, 3, 4, and 5 Gy at a rate of 1.1 Gy/min. Immediately after irradiation, cells were trypsinized using 0.05% trypsin-EDTA (Gibco^TM^, Thermo Fisher Scientific, France) and counted in order to seed 3 000 cells per well in 24-well plates (SPL Life Sciences, Deutscher, France) for each experimental condition, with four replicate wells per condition. Cell concentrations were measured using Countess cell counting chamber slides (C10228, Life Technologies). The plate is then incubated for 3 h to allow cell attachment, after which 2 mL of complete medium are added to each well to ensure sufficient nutrient availability during the time-lapse experiment. Subsequently, the plate is transferred to the microscope for live-cell imaging. An integrated incubation chamber (TokaiHit) on the microscope allows the cells to be kept at 37°C in a 5% CO_2_ atmosphere and 95% humidity. The epifluorescence microscope (Nikon Eclipse TS2R inverted microscope equipped with a Hamamatsu Orca Flash 4.0LT camera) acquires the images with a 10x magnification. Cells express Green Fluorescent Protein, the laser excitation wavelength is set to 400 nm and emission is set to 510 nm. For all regions of interest, manual focusing is performed using the CellSens software (Olympus, ScopPro, France) and is maintained throughout the time-lapse acquisition. The microscope acquires images of all regions of interest every 30 minutes over a period of 4 days, during which no phototoxic effects were observed.

### Analysis

**Fig 1:**
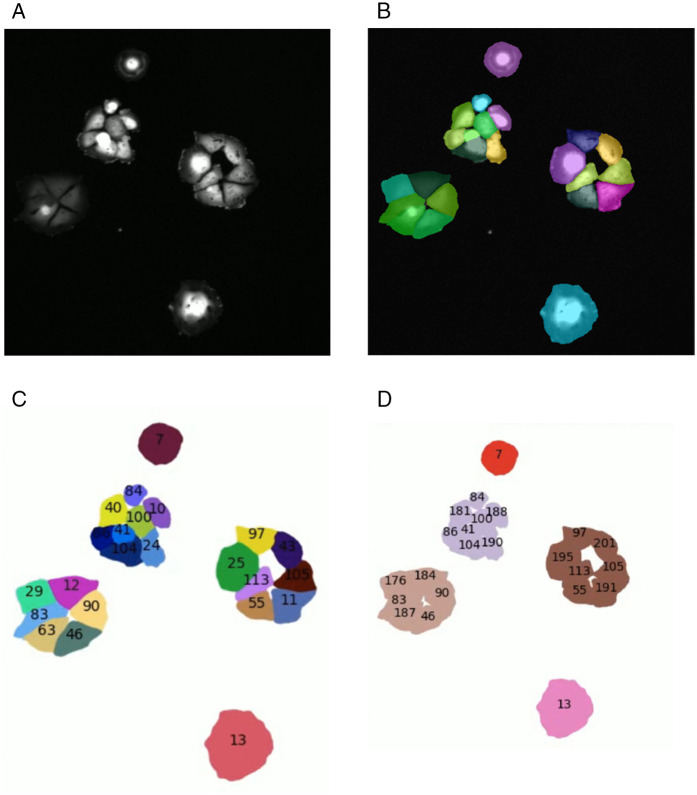
Visualization of the main analysis steps: (a) raw microscope image, (b) segmentation, (c) tracking, and (d) linking.

CellLineageTracking (CLT) algorithm is a custom single-cell tracking algorithm specifically designed for this specific experimental setup and imaging conditions, as the high frequency of cell divisions and the impact of irradiation required a tailored approach. The pipeline is organized into four main steps illustrated in Fig 1:

(a) **Image Pre-processing:** Raw microscopy images are first processed to reduce the background noise and adjust the contrast, ensuring optimal visualization of cellular structures, more informations are provided in S2 Appendix.
(b) **Segmentation:** Accurate cell segmentation is crucial, especially when cells are adjacent after division or show atypical morphologies induced by irradiation. For this purpose, customized models are trained based on the Cellpose machine learning framework [16,17], which represents the state of the art in cell segmentation [18,19,22]. Its flexibility and its ability to be trained on user-specific datasets allows the development of models tailored to each irradiation dose, thereby ensuring optimal segmentation performance in this study, more informations are provided in S2 Appendix.
(c) **Tracking:** Each segmented cell is assigned a unique identifier and tracked across successive frames. The tracking algorithm relies on minimizing variations in cell contours between consecutive images to ensure robust identity preservation over time. More informations are provided in S3 Appendix.
(d) **Linking:** After a new cell has been detected, its contours are compared with those in the previous frame to determine the corresponding mother cell. Cell division events are thereby detected, allowing the reconstruction of complete lineage trees. As illustrated in Fig 1, cells sharing the same color originate from the same initial mother cell. More informations are provided in S4 Appendix.

At the end of the pipeline execution, two outputs are generated. At each step, videos enables visual inspection of the results. A table is also exported, containing detailed single-cell information throughout the experiment. This includes, for each time point, cell position, surface area, and fluorescence intensity. This table also includes metadata describing the cell’s origin (e.g., initially present, daughter of a division, or entering from the image boundary) and its disappearance (e.g., present until the last frame, dividing into daughters, or leaving the field of view). These detailed information allow for rigorous filtering of cells used in subsequent analyses, ensuring the exclusion of artifacts such as cellular debris or edge-related artifacts. To handle the large volume of data, the 144 videos in this study were computed in parallel on the IN2P3 computing center. Each run lasted approximately 3 hours, of which 70% corresponds to segmentation.

The detailed CLT algorithm, including its implementation details and additional documentation, is provided in S1 Appendix.

For CCDF-based analyses, uncertainties were estimated using a Monte Carlo bootstrap approach [23]: empirical distributions were resampled 10^3^ times from 10^5^ values drawn from the observed CCDF, with 100 occurrences per resample, and the standard deviation of the resulting median distribution was used as the error estimate (the number of cells *N* per condition is reported). For all other figures, error bars correspond to the standard deviation of the measured values.

### Performance metrics

The performance of the CLT framework was evaluated using metrics assessing segmentation, tracking, and lineage reconstruction accuracy in heterogeneous irradiated mammalian cell populations. Segmentation quality was quantified using Detection Accuracy (DetA), which remained above 80% even at stringent IoU thresholds and showed stable performance over long-term acquisitions despite increasing morphological variability. Tracking consistency was assessed using Association Accuracy (AssA), exceeding 80% across irradiation doses and indicating robust temporal identity preservation. Because existing benchmarks do not adequately capture lineage reconstruction in heterogeneous irradiated mammalian systems, dedicated biologically driven metrics were introduced to evaluate lineage tree assignment, division detection, and cell lifetime reconstruction. This analysis prioritizes correct separation between lineage trees and accurate estimation of cell cycle duration, as these parameters directly support downstream biological categorization. Lineage assignment accuracy exceeded 95%, with remaining discrepancies mainly corresponding to spontaneous appearances excluded from biological analyses. Division detection and lifetime reconstruction also showed high agreement with ground truth, demonstrating that CLT reliably preserves lineage structure under irradiation-induced heterogeneity. Together, these results validate the robustness of the framework for long-term lineage analysis for this set-up, more informations are available in S6 Appendix.

### Parameters

- **Average population growth:** To capture the global dynamics of the cell population, the total number of cells as a function of time was fitted with an exponential growth model [24]:
  $$N(t)=N_{0}\;e^{rt},$$ (1)
  where *N*_0_ is the initial number of cells at time *t* = 0, and *r* is the growth rate. From Eq (1), the doubling time *T*_*d*_ is directly derived as
  $$T_{d}=\frac{\ln(2)}{r}.$$ (2)
  This formulation allows for a simple and direct comparison of proliferation kinetics between irradiated and control conditions, and relates them to literature.
- **Cell Surface Area:** The surface area of individual cells was obtained from the number of pixels belonging to the segmented region of the cell. Segmentation was performed on the GFP-labeled cytoplasm, which provides a reliable representation of the actual cell surface. Pixel counts were then scaled according to the microscope field of view (1 330 μm × 1 330 μm) to obtain physical units ($\mu {\text{m}}^{2}$).
- **Cell Diffusion Coefficient:** Individual cell diffusion was characterized during the full cell lifetime using the mean squared displacement (MSD) [25,26]:
  $$D\equiv \frac{\langle \Delta x^{2}+\Delta y^{2}\rangle }{\Delta t}$$ (3)
  For each cell, the barycenter coordinates (*x*,*y*) were extracted at every frame, and frame-to-frame displacements (Δx,Δy) were computed and normalized by the acquisition time interval Δt (30 min in this study) to obtain instantaneous diffusion estimates, with *D* denoting the effective diffusion coefficient ($\mu {\text{m}}^{2}$/h). To minimize the influence of extreme events (e.g., abrupt displacements during mitosis or rare tracking errors), the median value over the entire trajectory, rather than the mean, was used as the representative diffusion parameter. Although MSD is a simple statistical measure, it provides a robust approximation of cell diffusion by capturing overall displacement characteristics while minimizing the impact of erratic events.
- **Probability To Exceed (PTE):** To characterize the distribution of quantitative single-cell parameters, such as cell surface area or diffusion coefficient, PTE is defined as:
  PTE(x)=P(X≥x), (4)
  where *X* denotes the variable of interest [27]. By definition, the PTE starts at 1 and decreases monotonically toward 0 as *x* increases. This representation highlights differences in the distribution tails and allows for a direct estimation of the median value: it corresponds to the value of *x* where the PTE intersects a horizontal line at 0.5. To assess the statistical precision, error bars on the median were obtained using a Monte Carlo resampling procedure [23]. PTE is sometimes referred to as the Complementary Cumulative Distribution Function.

### Ethics statement

This study did not involve human participants, human data, or animal experimentation. All experiments were performed *in vitro* using established cancer cell lines. Therefore, ethical approval and informed consent are not required.

## Results

Videomicroscopy recordings of cultured cells were acquired following irradiation, with multiple videos obtained for each experimental condition, from multiples areas from different wells. This approach enables dynamic observation of cellular responses over time and allows for analysis of the effects of irradiation as a function of the delivered dose. One objective is to identify key biological parameters that are representative of the irradiation response and relevant for characterizing cellular behavior under these conditions. The maximum duration that can be analyzed for each experimental condition is inherently limited by cell proliferation, see S2 Appendix.

### From bulk population to individual cells

**Fig 2:**
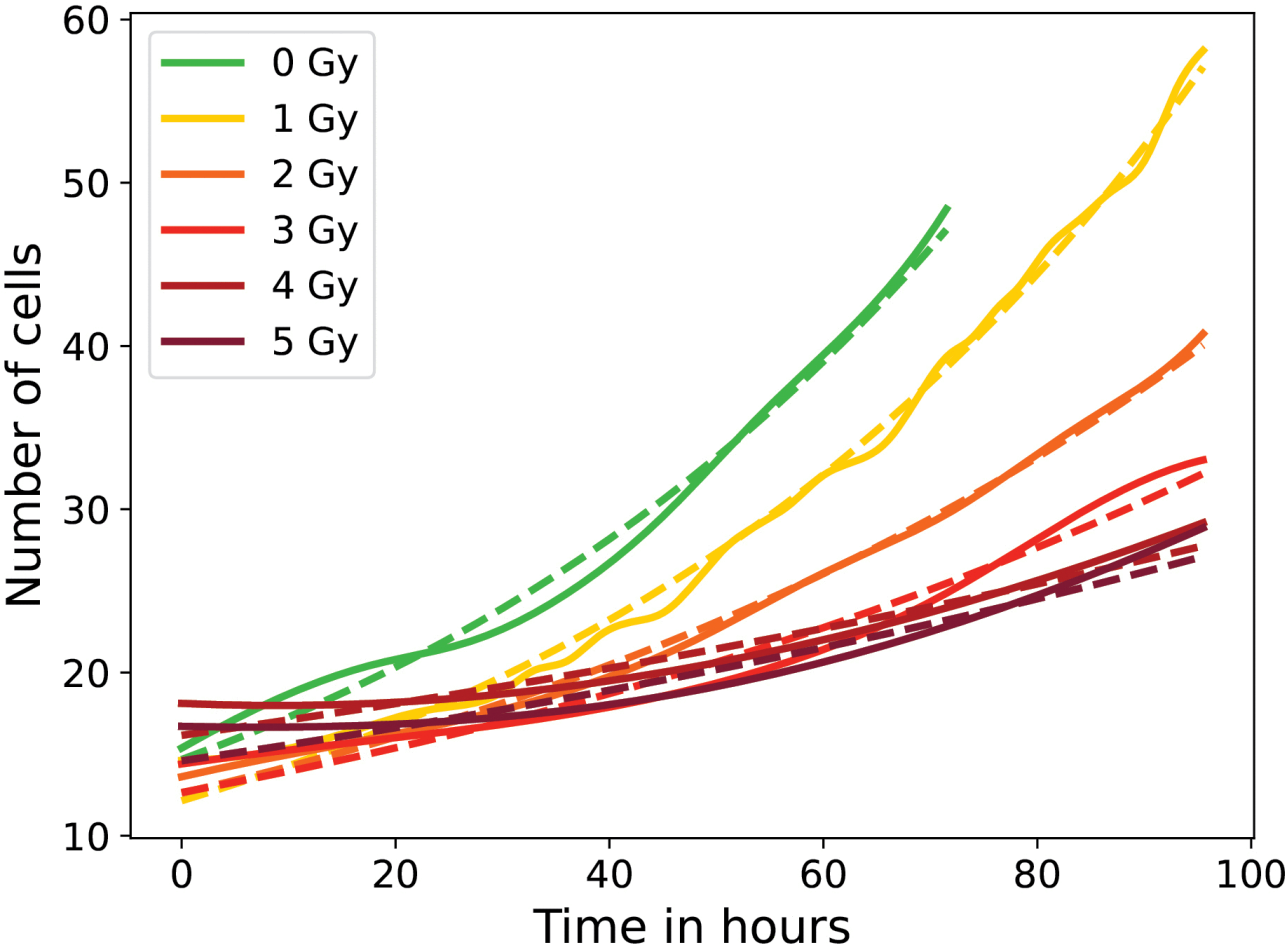
Evolution of the number of cells in one video over time for control and irradiated conditions. Exponential growth model fitted to estimate doubling times.

Among all biological parameters, the evolution of the number of cells over time, showed in Fig 2, is not the most representative parameter of the impact of X-ray irradiation (especially in this study with sub-lethal doses from 0Gy to 5 Gy), but often used as the main parameter [9,28]. However, the number of cells over time can be a first approximation of the effect of irradiation doses, particularly the doubling time of the cell population, which is linked to the cell division rate of the population.

**Fig 3:**
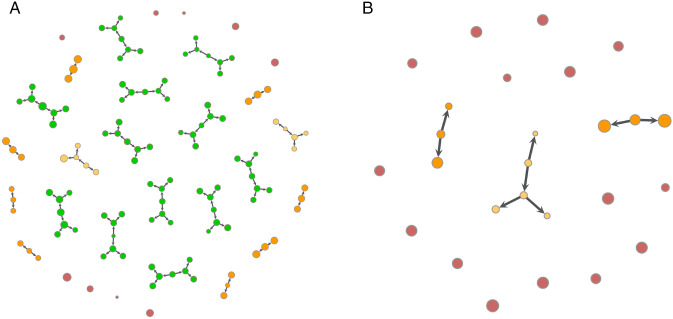
Lineage trees reconstructed with the CLT algorithm for representative wells under control and irradiated conditions. **(a)** Control sample (0 Gy) after 60 h and (b) irradiated sample (5 Gy) after 96 **h.** Each node represents an individual cell, with node size scaled to its measured surface area. Colors indicate tree size, allowing visualization of differences in clonal expansion between conditions.

For control cells, the doubling time of approximately *T*_*d*_ = 60 h was obtained, consistent with values reported in the literature [29]. The irradiated cells showed markedly slower proliferation, with *T*_*d*_ = 91 h at 2 Gy and *T*_*d*_ = 322 h at 5 Gy. To better understand the cellular response to irradiation, lineage trees were reconstructed for all cells in each experimental condition, using our CLT algorithm. These lineage trees provide a clear visualization of cell divisions and allow tracking of individual cell fates. Representative examples of reconstructed lineages for control (0 Gy) and irradiated (5 Gy) conditions are shown in Fig 3, providing an illustrative visualization. In the control condition, the number of initial cells is higher, and trees appear larger, more symmetric, and well developed. The uniformity in node size across successive divisions suggests that cell surface area remains relatively conserved throughout the cell divisions. In contrast, under the 5 Gy condition, only a few lineage trees undergo significant development. These trees are smaller and more asymmetric, with cells exhibiting markedly larger surface areas, as reflected by the larger node sizes. Overall, this representation serves as an illustrative overview, offering a first glimpse of the distinct lineage trees categories that will be analyzed in detail in the subsequent sections.

**Fig 4:**
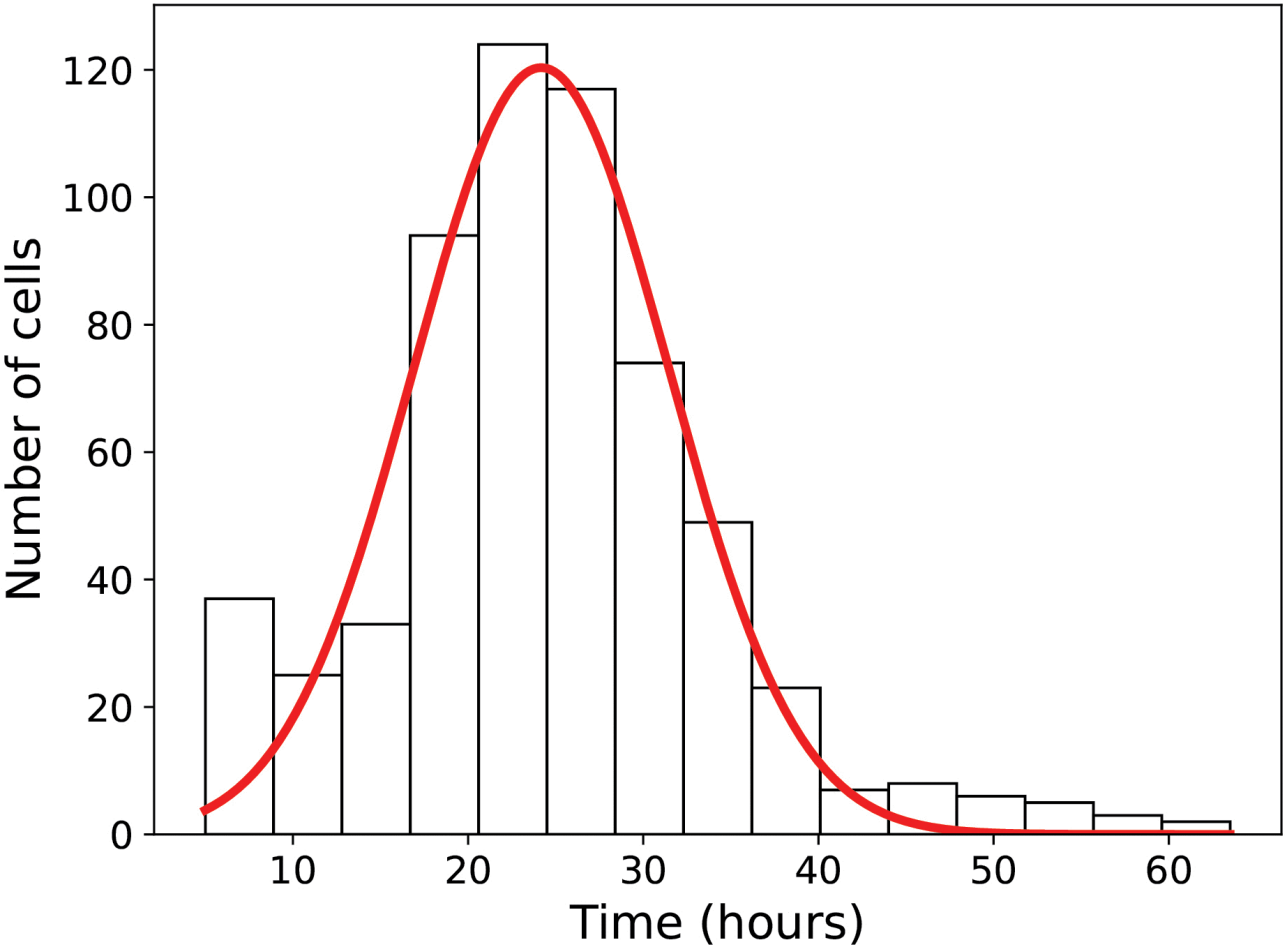
Cell cycle duration for control condition. Histogram in black and gaussian fit in red (N = 479 cells; Median = 24 h; Std = 9 **h)**. Only cells for which both the mother and daughters were identified were included.

Before investigating the impact of irradiation on the cell cycle, complete cell cycles under control conditions were analysed. Focusing on samples for which both the mother and daughter cells could be identified, entire cell cycles are tracked within the recorded movie. The distribution of the durations of the cell cycles shown in Fig 4. It follows an approximately Gaussian shape centered at 23 h, with distribution tails extending from 10 h to 40 h.

In this study, a “normal” cycle under control conditions is defined as duration between 10 h and 40 h, with the 40 h criterium set as the maximum duration of a normal cell cycle for subsequent analyses, see S2 Appendix.

### Lineage trees categories

Lineage trees were categorized according to the behavior of their initial cell, and three main categories were defined according to the duration of the cell cycle.

- **Proliferative cell trees**, in which the initial cell continued to divide with dynamics comparable to control cells, corresponding to cell cycle durations below the 40 h threshold.
- **Transiently Arrested (TA) cell trees**, in which the initial cell experienced an extended cycle arrest before resuming proliferation, corresponding to initial cell cycle durations above the 40 h threshold. Within this category, two subcategories are distinguished for the cells of these trees:
  - **Initial Transiently Arrested (ITA) cells**, the initial cell of the transiently arrested trees
  - **Re-Proliferating Transiently Arrested (RPTA) cells**, the progeny cells from the transiently arrested trees.

This distinction is motivated by observed differences in key bioparameters as will be shown further on.

- **Long-Term Arrested (LTA) cell trees**, in which the initial cell exited the cell cycle, within the window of 4 days. Strictly speaking, this category does not represent a tree, since it contains single cell.

To rigorously assess the impact of irradiation, cells were trypsinized and plated at equal densities across all experimental conditions. This uniform seeding enables the calculation of plating efficiency as a function of radiation dose by quantifying the number of cells present at the start of each experiment. For comparison purposes, the values were normalized to the control condition, which was set to 100%. The distribution of cell categories changed markedly over the observation period in a dose-dependent manner. Initially, proliferative cells dominated at low doses, while ITA/RPTA/LTA cells were more prevalent at higher doses. Over time, proliferative cells expanded at all doses, though their relative increase decreased with higher irradiation, and ITA/RPTA/LTA cells remained significant at intermediate and high doses, as summarized in Table 1.

**Table 1:** Initial cell numbers and cumulative cell counts across irradiation doses.

Dose (Gy)	Initial plating cells at *t* = 0 (total wells)	Initial cells at *t* = 0 (all films)	Proliferating (∀t)	ITA (∀t)	RPTA (∀t)	LTA (∀t)
0	6 574 (100%)	326 (27 movies)	1 292	23	90	62
1	5 312 (80%)	400 (33 movies)	683	22	84	70
2	4 724 (72%)	363 (33 movies)	616	39	170	145
3	4 507 (69%)	394 (34 movies)	526	68	272	230
4	5 263 (80%)	514 (36 movies)	232	95	362	314
5	4 967 (76%)	473 (36 movies)	274	69	258	305
Total	31 347	2 570	3 644	317	1 238	1 148

Initial plating corresponds to the total number of cells present at *t* = 0, pooled across the four wells associated with each dose. Initial cells across all films correspond to the total number of cells detected at the first frame of all movies acquired for a given dose. Proliferating, ITA, RPTA and LTA report cumulative counts of unique cells over all time points (∀t).

**Fig 5:**
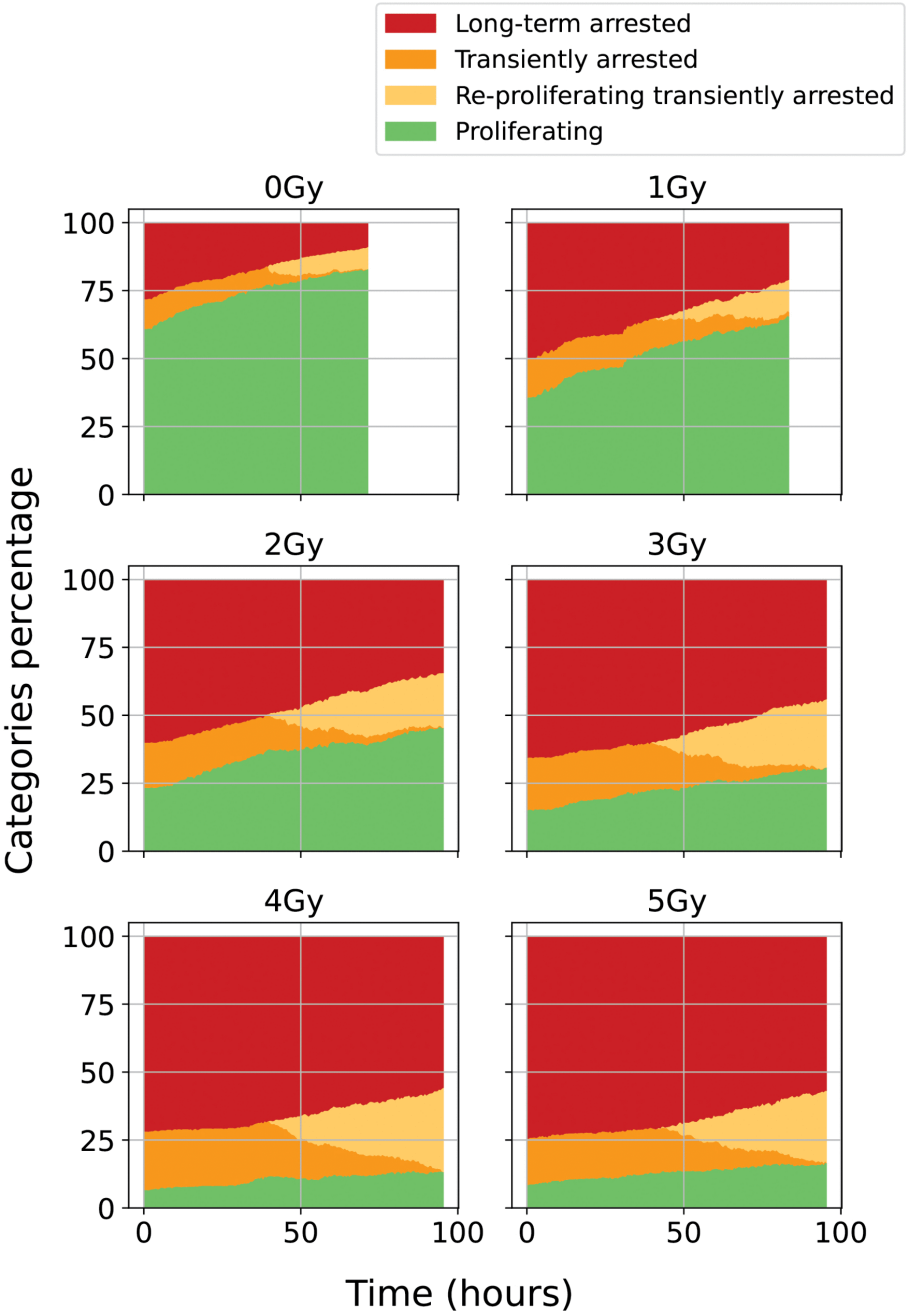
Classification of cells over time into the different categories for control condition 0 Gy and irradiated conditions (1 to 5 Gy). Proliferative cells in green, transiently arrested trees in orange (ITA cells in dark orange, RPTA in light orange), and LTA cells in red.

The temporal distribution of these four cell categories for each irradiation dose is presented in Fig 5.

For the control condition (0 Gy), only a very small fraction of the population was classified as ITA, RPTA, or LTA cells. These rare cases reflect naturally occurring variations rather than irradiation-induced effects, and their numbers are therefore too limited to provide meaningful statistics in the following analysis. Instead, the results for the control condition will primarily be interpreted based on the proliferative trees, which represent the vast majority of cells.

In the following sections, the results are presented separately for the three categories of trees: proliferative, transiently arrested, and LTA. For each category, analysis of both individual cell surface area and diffusion behavior are detailed.

### Proliferative cell trees

**Fig 6:**
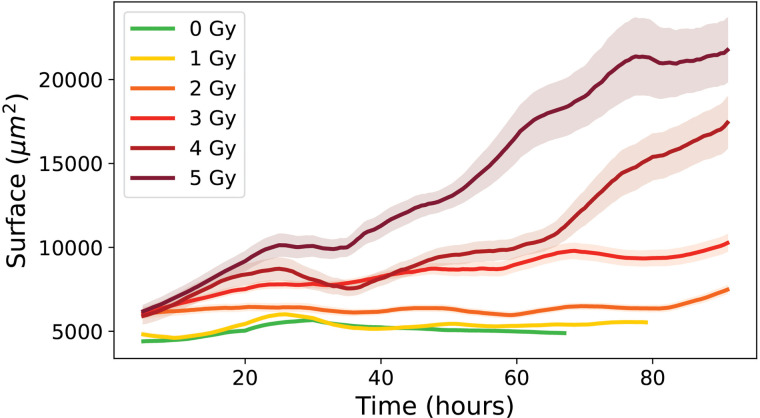
Evolution of the mean individual surface area over time for control condition 0 Gy and irradiated conditions (1 to 5 Gy), for cells from proliferative cell trees. Errors bars represent this standard deviation of the cell population.

First, the evolution of cell surface area over time was examined for proliferative cell trees, showed in Fig 6. In control conditions (0 Gy), cell surface area stabilized after several hours. The surface area increased with time and with radiation dose. At 0 Gy, the mean surface was relatively low (4 586 ± 47 $\mu m^{2}$), but it rose steadily up to 3 Gy (10 262 ± 495 $\mu m^{2}$). At higher doses (4 Gy and 5 Gy), the mean surface area remained high (17 430 ± 1 515 $\mu m^{2}$ and 21 755 ± 1 899 $\mu m^{2}$, respectively), although with considerable variability, as reflected by the standard deviations.

**Fig 7:**
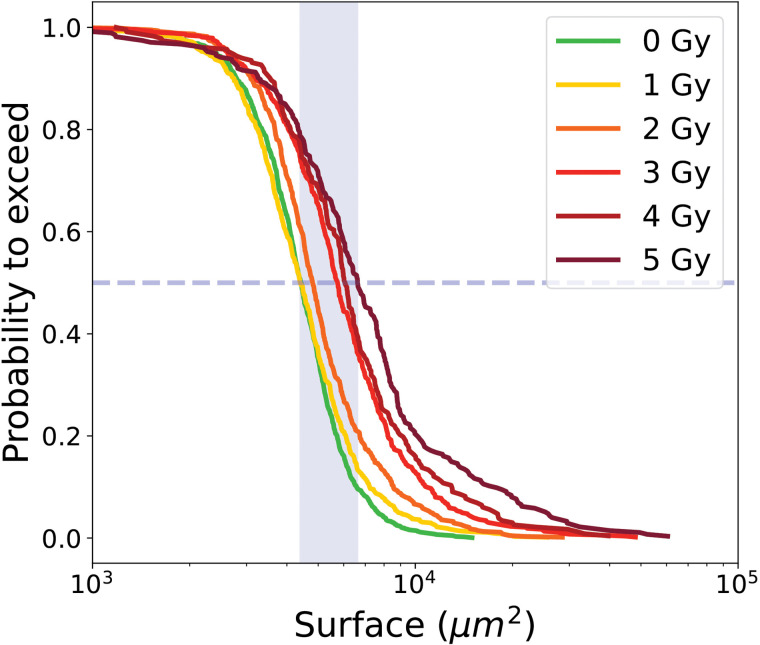
PTE of surface area of cells from proliferative cell trees for control condition 0 Gy and irradiated conditions (1 to 5 Gy). Dashed line indicate the median value at the intersection with the curve, while the blue shaded area represents the range of median values across all conditions.

Fig 7 shows the PTE of cell surface area for proliferative trees and Table 2 summarizes the medians of the PTE as a function of radiation dose, together with the corresponding error bars and sample sizes. Error bars indicate statistical variability and are estimated by Monte Carlo resampling.

**Table 2:** Surface area and diffusion coefficient of proliferating cells.

Dose (Gy)	Surface median ($\mu {\text{m}}^{2}$)	Diffusion median ($\mu {\text{m}}^{2}$/h)
0	4 402 ± 175	0.375 ± 0.064
1	4 399 ± 220	0.312 ± 0.047
2	4 805 ± 188	0.330 ± 0.047
3	5 738 ± 267	0.255 ± 0.042
4	6 037 ± 233	0.213 ± 0.028
5	6 620 ± 442	0.455 ± 0.127

Median values of individual cell surface areas and diffusion coefficients for proliferating cell trees under control (0 Gy) and irradiated conditions (1–5 Gy).

The median surface area of cells from proliferative trees gradually increased with radiation dose, ranging from 4 402 ± 175 $\mu {\text{m}}^{2}$ at 0 Gy to 6 620 ± 442 $\mu {\text{m}}^{2}$ at 5 Gy.

**Fig 8:**
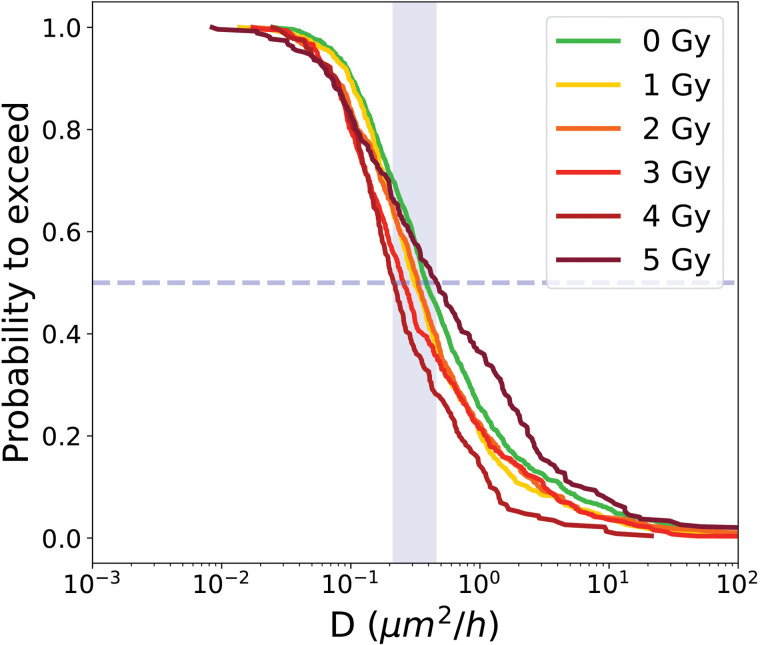
PTE of diffusion coefficient of cells from proliferative cell trees for control condition 0Gy and irradiated conditions (1 to 5 Gy). Dashed line indicate the median value at the intersection with the curve, while the blue shaded area represents the range of median values across all conditions.

Quantifying cell diffusion at the single-cell level is challenging, as in most studies motility is estimated at the population level, yielding an average value that masks cell-to-cell variability. Here, by quantifying diffusion at the individual level, the full heterogeneity of diffusion is captured within each condition. To further characterize the distribution of diffusion, the PTE of the diffusion coefficient is computed, showed in Fig 8.

For proliferative cells, the median diffusion coefficients are summarized in Table 2. Error bars indicate statistical variability and are estimated by Monte Carlo resampling.

The median diffusion coefficients of cells from proliferative trees ranged from 0.213 ± 0.028 $\mu {\text{m}}^{2}$/h to 0.455 ± 0.127 $\mu {\text{m}}^{2}$/h across the different irradiation doses, with no clear trend apparent due to the overlap of error bars.

In conclusion, in this section, the median surface area of proliferative cells is observed to increase progressively with radiation dose, while the median diffusion coefficients vary across doses without displaying a clear trend.

### Transiently arrested cell trees

**Fig 9:**
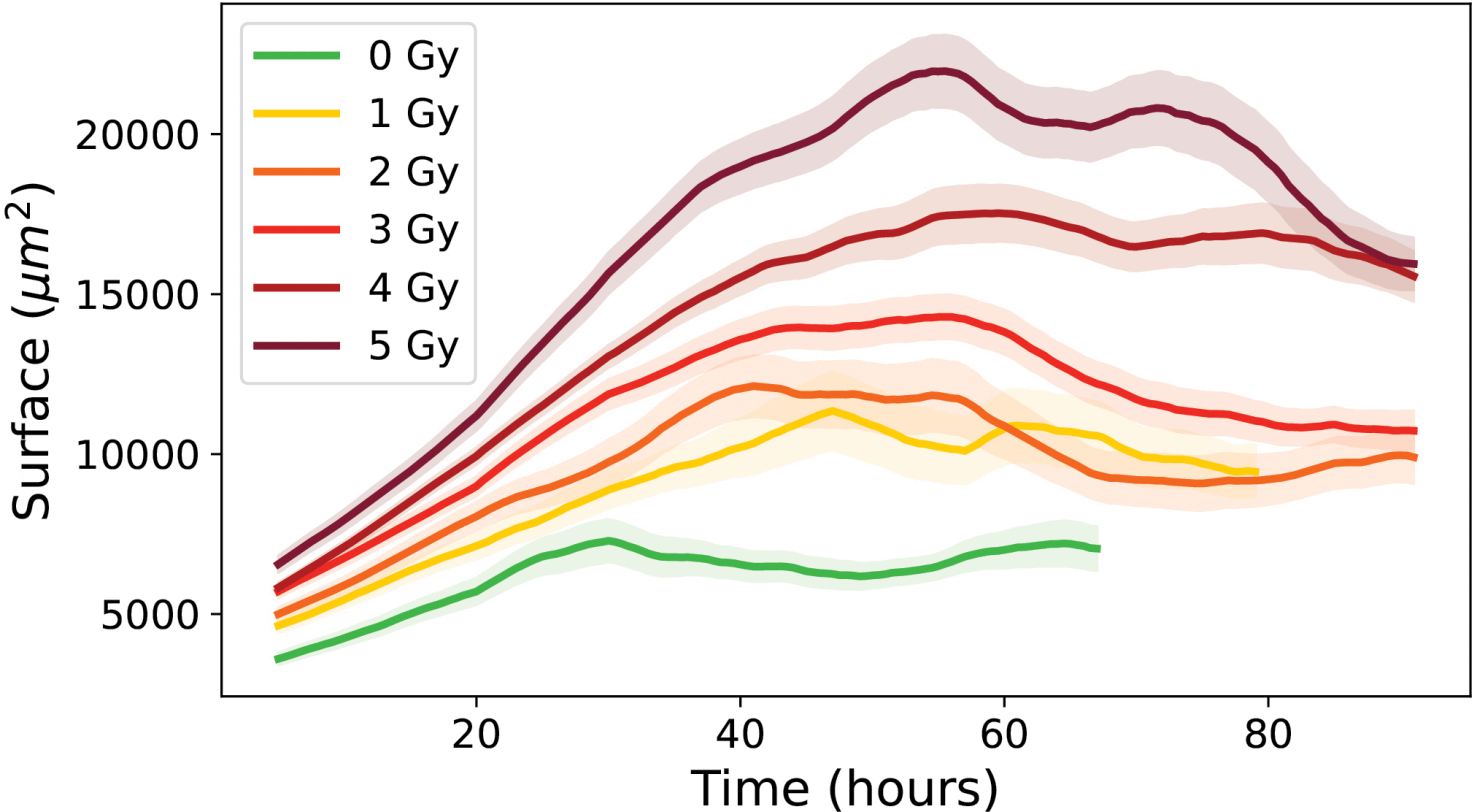
Evolution of the mean individual surface over time for control condition 0Gy and irradiated conditions (1 to 5 Gy); for cells from transliently arrested cell trees. Errors bars represent this standard deviation of the cell population.

As shown in Fig 9, cells from transiently arrested cell trees showed an initial increase in surface area, followed by a decrease and subsequent stabilization.

At 0 Gy, the mean surface area reached 7 044 ± 688 $\mu {\text{m}}^{2}$ after 3 days, and remained high across all doses, with values from 9 902 ± 829 $\mu {\text{m}}^{2}$ at 2 Gy to 15 944 ± 811 $\mu {\text{m}}^{2}$ at 5 Gy after 4 days. The associated standard deviations represent substantial variability between individual cells.

**Fig 10:**
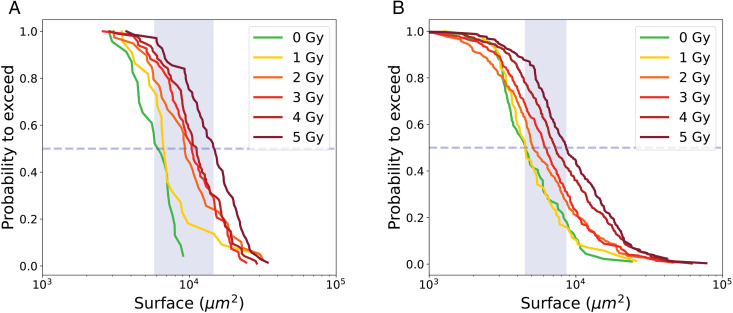
PTE of surface area of cells from transiently arrested trees, for control condition 0Gy and irradiated conditions (1 to 5 Gy); (a) ITA cell, (b) RPTA cells. Dashed line indicate the median value at the intersection with the curve, while the blue shaded area represents the range of median values across all conditions.

The PTE analysis of surface areas for transiently arrested trees, distinguishing ITA and RPTA cells, is shown in Fig 10 and summarized in Table 3.

**Table 3:** Surface area and diffusion coefficient of transiently arrested cell trees.

	ITA cells		RPTA cells	
Dose (Gy)	Surface ($\mu {\text{m}}^{2}$)	Diffusion ($\mu {\text{m}}^{2}$/h)	Surface ($\mu {\text{m}}^{2}$)	Diffusion ($\mu {\text{m}}^{2}$/h)
0	5 800 ± 413	0.081 ± 0.016	4 534 ± 347	0.636 ± 0.119
1	6 612 ± 129	0.080 ± 0.010	4 510 ± 259	0.741 ± 0.110
2	9 303 ± 459	0.045 ± 0.008	5 079 ± 535	0.459 ± 0.098
3	10 358 ± 619	0.048 ± 0.004	6 184 ± 464	0.430 ± 0.100
4	11 152 ± 593	0.041 ± 0.004	7 211 ± 682	0.707 ± 0.144
5	14 553 ± 983	0.043 ± 0.005	8 580 ± 910	0.587 ± 0.126

Median values of individual cell surface areas and diffusion coefficients for transiently arrested cell trees (1–5 Gy).

The median surface areas of transiently arrested trees increased with irradiation dose for both ITA and RPTA cells, ranging from 5 800 ± 413 $\mu {\text{m}}^{2}$ to 14 553 ± 983 $\mu {\text{m}}^{2}$ for ITA cells and from 4 510 ± 259 $\mu {\text{m}}^{2}$ to 8 580 ± 910 $\mu {\text{m}}^{2}$ for RPTA cells. RPTA cells consistently exhibit lower surface areas than ITA cells at the same irradiation dose.

**Fig 11:**
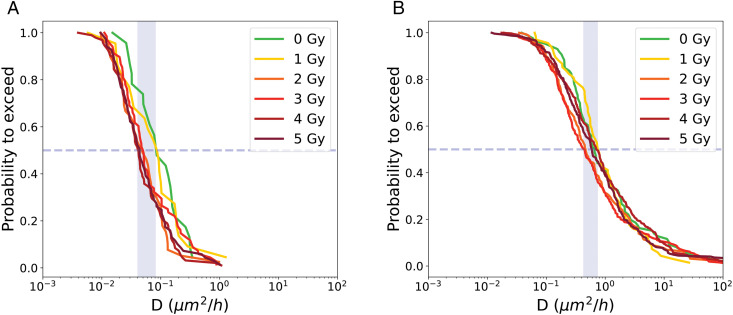
PTE of diffusion coefficient of cells from transiently arrested cell trees for control condition 0Gy and irradiated conditions (1 to 5 Gy). **(a)** ITA cell, **(b)** RPTA cell. Dashed line indicate the median value at the intersection with the curve, while the blue shaded area represents the range of median values across all conditions.

The diffusion coefficient analysis provided complementary information (Fig 11, Table 3). ITA cells showed very low median diffusion coefficients, with values between 0.041 $\mu {\text{m}}^{2}$/h at 4 Gy and 0.080 $\mu {\text{m}}^{2}$/h at 1 Gy. In contrast, RPTA cells exhibited consistently higher values, ranging from 0.430 $\mu {\text{m}}^{2}$/h at 3 Gy to 0.741 $\mu {\text{m}}^{2}$/h at 1 Gy. Sample sizes were substantially larger for secondary cells (from *N* = 84 at 1 Gy to *N* = 258 at 5 Gy) compared to primary cells (from *N* = 22 at 1 Gy to *N* = 69 at 5 Gy).

The median diffusion coefficients of transiently arrested trees do not show a clear trend with increasing irradiation dose within each category. However, at the same irradiation dose, RPTA cells consistently exhibit higher diffusion than ITA cells.

In conclusion, in this section, ITA and RPTA cells show an initial increase in surface area followed by stabilization at relatively high values across all irradiation doses. The median surface area rises with dose for both ITA and RPTA cells, the latter consistently exhibiting smaller surfaces. The median diffusion coefficients show no clear dose-dependent trend within each category, although RPTA cells display higher diffusion values than ITA cells at the same dose.

### Long-term arrested cell

**Fig 12:**
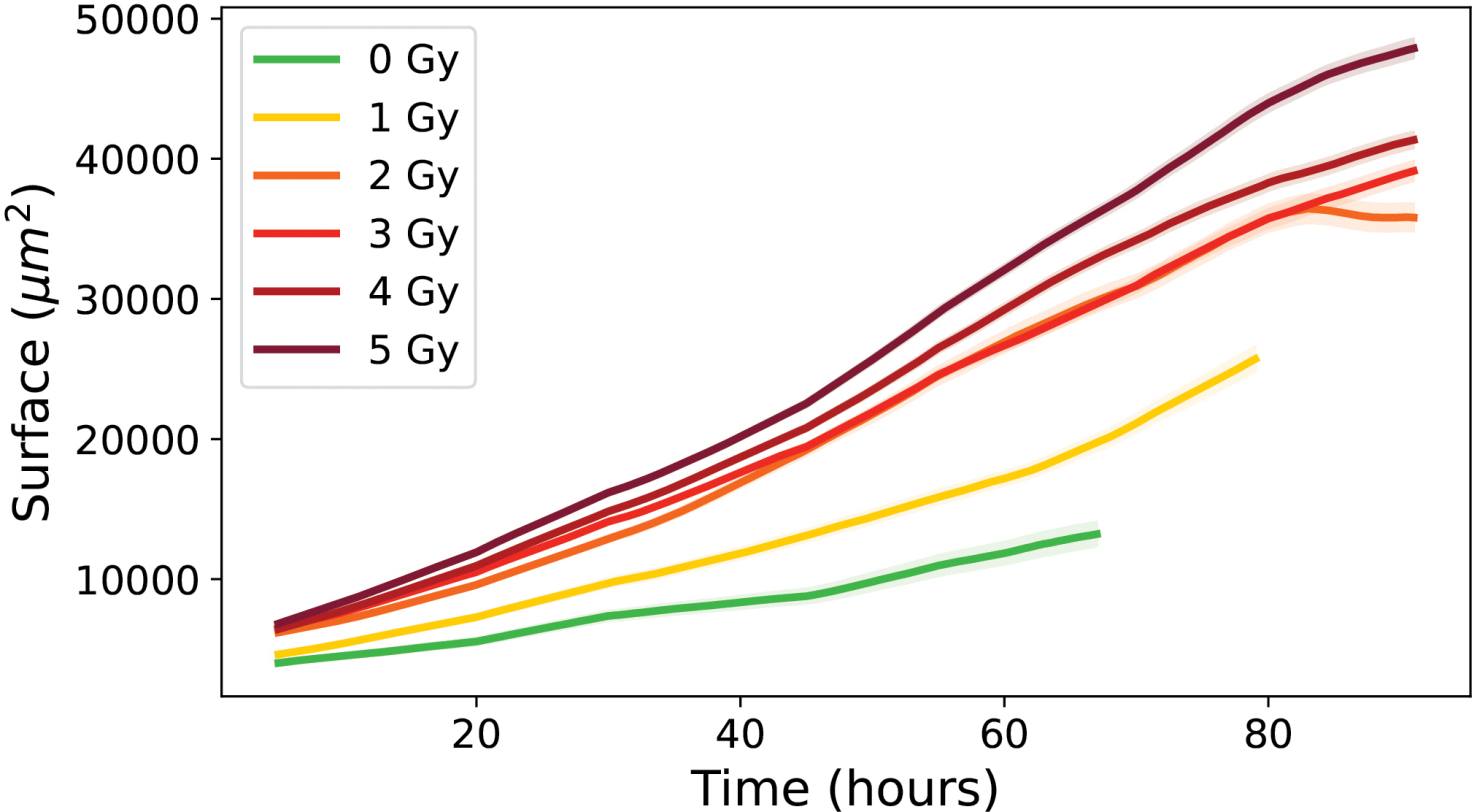
Evolution of the mean individual surface area over time for irradiated conditions (1 to 5 Gy) for LTA cells. Errors bars represent this standard deviation of the cell population.

As shown in Fig 12, LTA cells revealed a continuous increase in surface area throughout the observation window, systematically higher than cells in other categories.

The mean surface were consistently high for all irradiated conditions, and the standard deviations indicated a high level of variability among individual cells.

**Fig 13:**
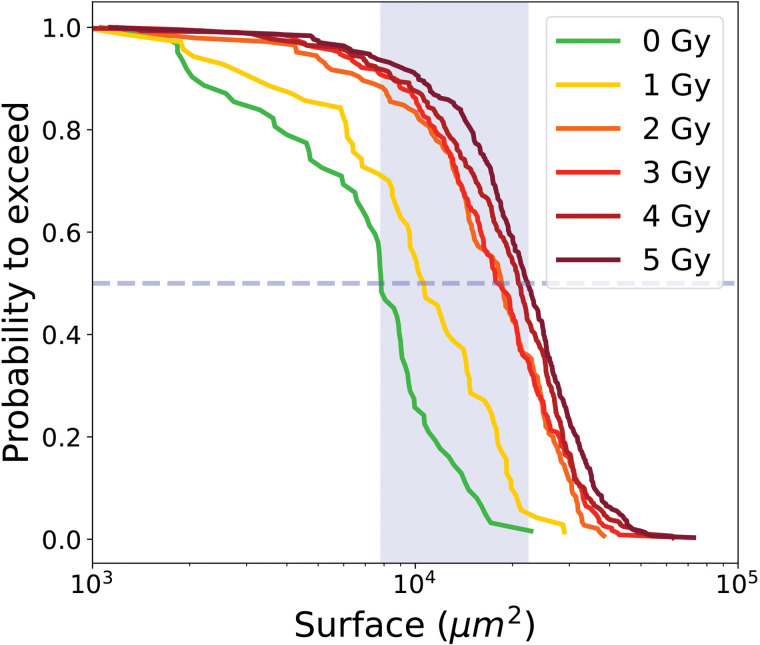
PTE of surface area of LTA cells for control condition 0Gy and irradiated conditions (1 to 5 Gy). Dashed line indicate the median value at the intersection with the curve, while the blue shaded area represents the range of median values across all conditions.

The probability to exceed (PTE) of individual cell surface areas is showed in Fig 13 and the median values are summarized in Table 4. The median surface areas of LTA cells at doses 2, 3, 4, and 5 Gy were very similar, ranging from 18 483–22 297 $\mu {\text{m}}^{2}$, while the median values at 0Gy and 1 Gy were lower at 7 827 $\mu {\text{m}}^{2}$ and 10 480 $\mu {\text{m}}^{2}$ respectively. Error bars, obtained by Monte Carlo resampling, highlight variability in the distribution but remains within a narrow range compared to the absolute surface values.

**Table 4:** Surface area and diffusion coefficient of LTA cells.

Dose (Gy)	Surface median ($\mu {\text{m}}^{2}$)	Diffusion median ($\mu {\text{m}}^{2}$/h)
0	7 827 ± 403	0.030 ± 0.004
1	10 480 ± 851	0.026 ± 0.002
2	18 483 ± 1 311	0.022 ± 0.003
3	17 856 ± 1 330	0.025 ± 0.003
4	20 851 ± 1 121	0.022 ± 0.002
5	22 297 ± 1 333	0.023 ± 0.002

Median values of individual cell surface areas and diffusion coefficients for long-term arrested (LTA) cells (0–5 Gy).

**Fig 14:**
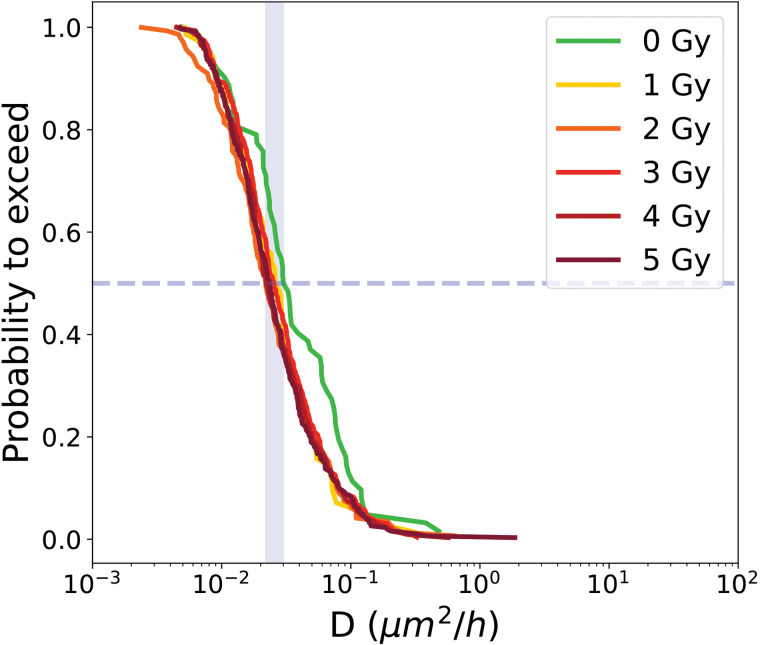
PTE of diffusion coefficient of LTA cells for control condition 0Gy and irradiated conditions (1 to 5 Gy). Dashed line indicate the median value at the intersection with the curve, while the blue shaded area represents the range of median values across all conditions.

Analysis of diffusion confirmed the distinct behavior of LTA cells. The PTE of diffusion coefficients (Fig 14) shows consistently low values across all conditions. Median diffusion coefficients remained within a narrow range, from 0.22 $\mu {\text{m}}^{2}$/h at 4 Gy to 0.390$\mu {\text{m}}^{2}$/h at 0 Gy, as reported in Table 4.

The number of LTA cells contributing to this analysis was substantial across doses, with sample sizes between *N* = 62 and *N* = 305. Although error bars, calculated by Monte Carlo resampling, partly overlapped between doses, the overall distribution consistently indicated very low diffusion values for LTA cells.

In conclusion, in this section, LTA cells show a continuous increase in surface area over time, reaching higher values than other cell categories across all irradiation doses. The median surface areas remain similar from 2 Gy to 5 Gy, with limited variability relative to their absolute values. Diffusion analysis reveals uniformly low coefficients across all conditions, with minimal variation between doses and consistently small error bars.

## Discussion

The cell cycle duration for control condition (Fig 4) is a benchmark to assess the purity of our cell tree reconstruction. Indeed, of all the conditions, the control condition remains the most difficult to compute due to the large number of proliferating cells, creating challenging clusters. The fact that the outliers of this distribution are rare indicates a high quality of trees reconstruction (>95%). After visual inspection of these outliers, it was concluded that cell cycles shorter than 10 hours always corresponded to abnormal cases in the analysis, which were excluded from our study (<5%), see S2 Appendix. Cell cycles exceeding 40 hours reflected a genuine biological slowdown observed in a few cells under control conditions, this criterium was therefore used to define the different cell categories. The median cell cycle duration of approximately 24 h, is consistent with previously reported values in the literature [30]. Additionally, several studies have reported the presence of distinct cellular phenotypes within a single MCF7 population [31,32], highlighting intrinsic heterogeneity in cell cycle duration.

The evolution of the number of cells in each category in Fig 5 reflect the biological response of cells to irradiation. At low doses, most cells proliferate, whereas higher doses induce cell cycle arrest or senescence in a dose-dependent manner, activating growth-inhibitory mechanisms that prevent the propagation of damaged cells [4,33].

**Fig 15:**
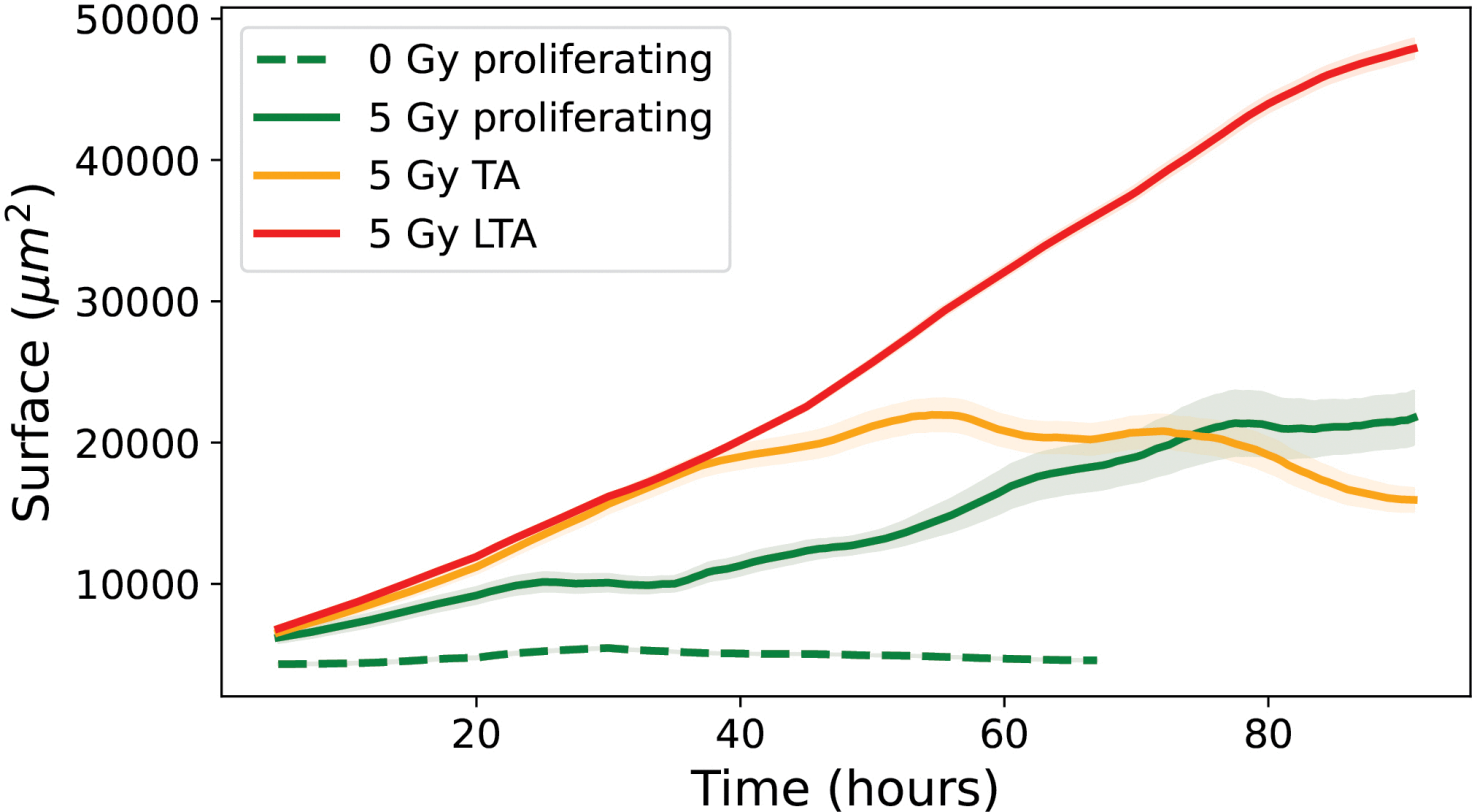
Evolution of the mean individual surface area over time of control for proliferative cells (dashed line) and 5 Gy irradiated conditions for all cells categories (solid line): proliferative, transiently arrested (TA) and long-term arrested (LTA). Errors bars represent this standard deviation of the cell population.

Fig 15 shows the evolution of cell surface area over time for the three categories (proliferative, transiently arrested and long-term arrested) highlighting only proliferative cells at 0 Gy and all categories at 5 Gy, thus allowing a direct comparison of surface areas across conditions and cell categories. Only the 5 Gy condition is displayed to preserve figure readability; however, similar trends were observed across all irradiation doses. For proliferative cells at 0 Gy, the surface area remains essentially constant, with a slight initial increase due to cell seeding and adhesion, reaching around 4 586 ± 47 $\mu {\text{m}}^{2}$, reflecting the steady growth and division typical of healthy, unstressed cells [34]. At 5 Gy, proliferative cells exhibit a substantial increase over time, starting from an initial mean of 6 196 ± 346 $\mu {\text{m}}^{2}$ and reaching a final mean of 21 755 ± 1 899 $\mu {\text{m}}^{2}$, suggesting that surviving cells continue to grow despite irradiation-induced stress, possibly as a compensatory hypertrophy. Transiently arrested (TA) trees at 5 Gy begin at 6 557 ± 255 $\mu {\text{m}}^{2}$, show a transient peak during the observation period, and stabilize at a final mean of 15 944 ± 811 $\mu {\text{m}}^{2}$, consistent with temporary cell cycle arrest allowing growth without division, followed by repair mechanisms that restore a surface phenotype closer to that of control cells [35]. Long-term arrested (LTA) cells at 5 Gy start at 6 827 ± 63 $\mu {\text{m}}^{2}$ and show a continuous increase over time, reaching a final mean of 47 878 ± 687 $\mu {\text{m}}^{2}$, indicative of pronounced hypertrophy associated with senescence and the accumulation of cellular stress markers [4]. These observations highlight the differential responses of cells under irradiation: long-term arrested cells undergo maximal enlargement reflecting permanent growth arrest, transiently arrested cells show intermediate enlargement with partial recovery, and proliferative cells maintain moderate growth while preserving their division capacity [36].

**Fig 16:**
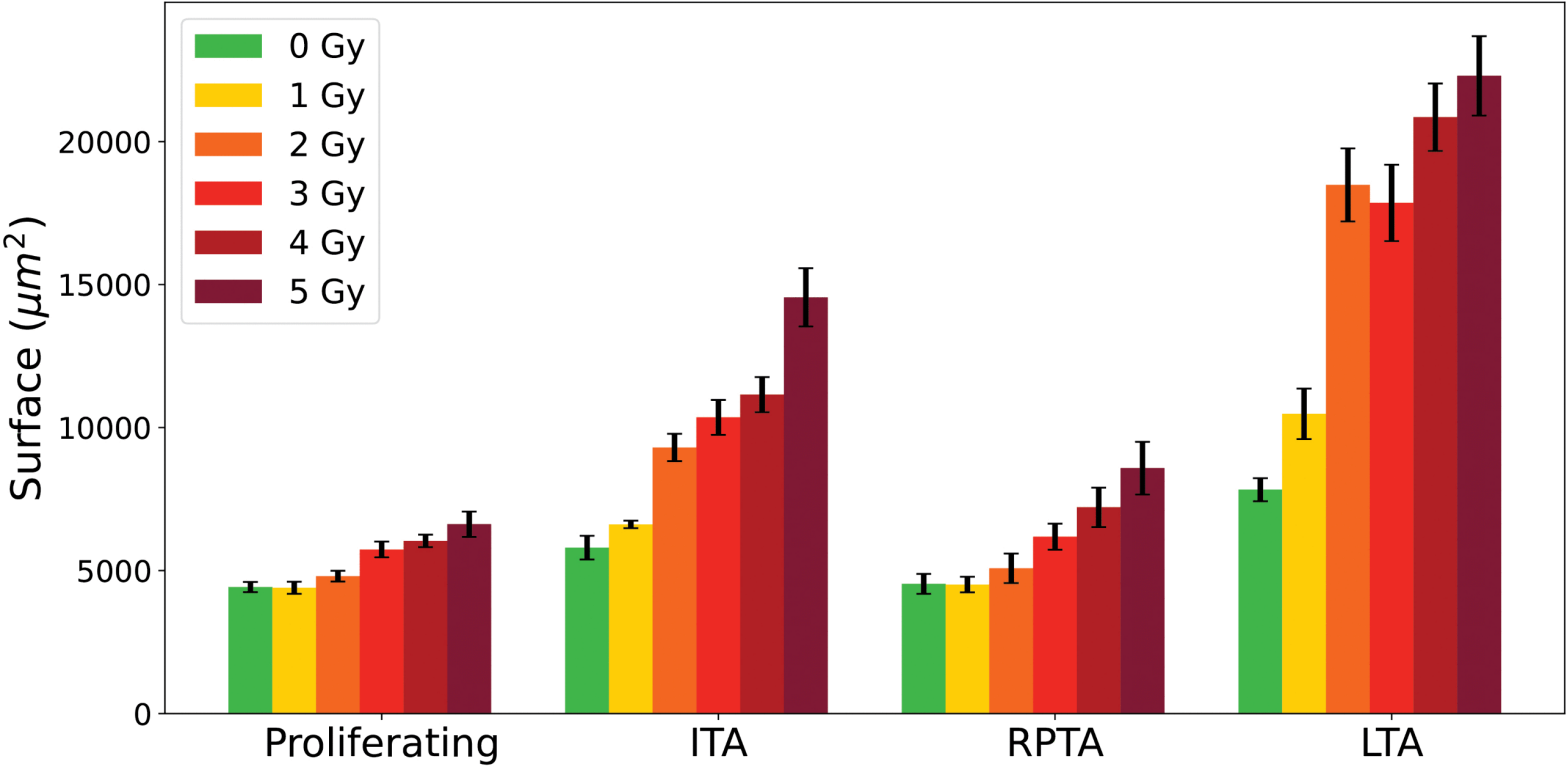
Median of individual cell surface area for proliferative cells, Initial Transliently Arrested (ITA) cells and Re-Proliferating Transiently Arrested (RPTA) cell, and Long-Term Arrested (LTA) cells from 0 to 5 Gy. Error bars are estimated using Monte Carlo resampling, see Materials and Methods.

Fig 16 shows the median values of the PTEs of cell surface area with error bars for all the different categories. There is a progressive increase in median surface area: medians for proliferative cells range from 4 402–6 620 $\mu {\text{m}}^{2}$, for initial transiently arrested cells (ITA) cells from 5 580–14 553 $\mu {\text{m}}^{2}$, for re-proliferating transiently arrested (RPTA) cells from 4 510–8 580 $\mu {\text{m}}^{2}$, and for long-term arrested (LTA) cells from 7 827–22 297 $\mu {\text{m}}^{2}$. This trend highlights the larger size of long-term arrested cells, consistent with their hypertrophic phenotype, while transiently arrested (TA) cells are intermediate, likely reflecting temporary cell cycle arrest that allows growth without division. Proliferative cells maintain the smallest median surface, reflecting balanced growth and division. The median values of RPTA cells almost coincides with the curve of proliferative cells, suggesting that these cells have undergone repair processes that restore behavior closer to the proliferative phenotype [35]. Finally, within each category, an increase in surface area is observed with increasing irradiation dose, which is particularly pronounced for transiently arrested and LTA cells. The error bars for transiently arrested cells, and even more so for LTA cells, are larger than those of proliferative cells, reflecting more heterogeneity within these populations. All pairwise comparisons performed, both between doses within each cell category (e.g., 0 Gy vs 5 Gy or 1 Gy vs 5 Gy) and between different cell categories at 5 Gy, yielded p-values less than 0.01, indicating that all observed differences were statistically significant. This observation indicates that confluence (i.e., the percentage of the field of view covered by cells) is not a reliable parameter for assessing the effects of *in vitro* irradiation. Relying on confluence introduces a bias, as it tends to underestimate the apparent lethality of the treatment.

**Fig 17:**
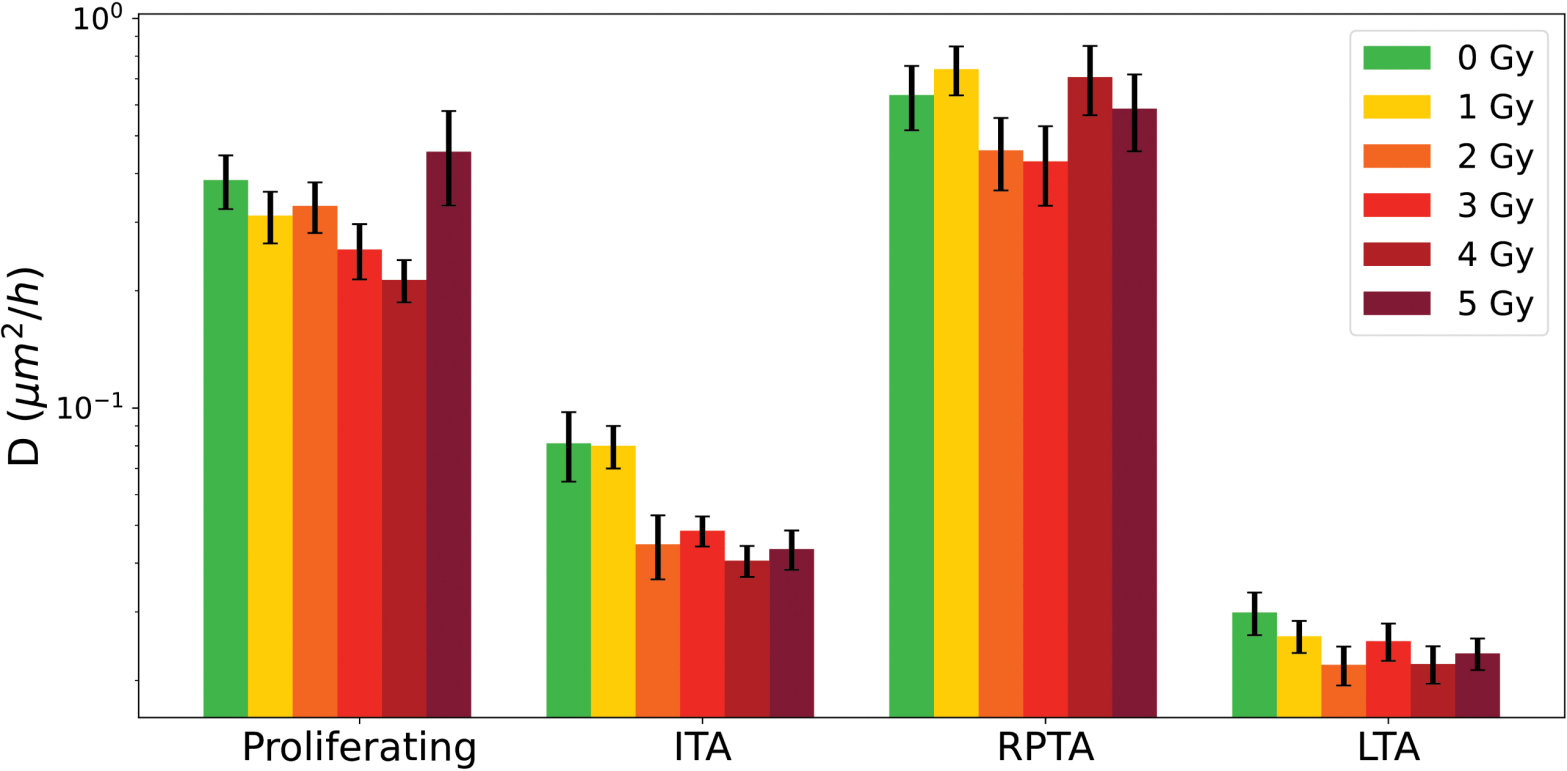
Median of individual cell diffusion coefficient for proliferative cell trees, Initial Transliently Arrested (ITA) cells and Re-Proliferating Transiently Arrested (RPTA) cell, and Long-Term Arrested (LTA) cells from 0 to 5 Gy. Error bars are estimated using Monte Carlo resampling, see Materials and Methods.

Fig 17 shows the median values of the PTEs of cell diffusion coefficient with error bars for all the different categories. As for the cell surface area differenciation between categories, cell diffusion coefficient are significantly different between categories. Proliferative cells exhibit the highest diffusion, with median values ranging from 0.213 to 0.455 $\mu {\text{m}}^{2}$/h across doses, consistent with active motility associated with ongoing proliferation [37]. ITA cells show markedly lower diffusion, ranging from 0.41 to 0.81 $\mu {\text{m}}^{2}$/h, whereas RPTA cells show diffusion values closer to those of proliferative cells (0.430 to 0.741$\mu {\text{m}}^{2}$/h), indicating recovery and a return toward a proliferative-like motility profile. LTA cells consistently exhibit the lowest diffusion (0.022 to 0.030 $\mu {\text{m}}^{2}$/h), reflecting their enlarged, stiffened morphology [4].

Unlike the cell surface area, within each cell category the effect of radiation dose on diffusion remains modest. For proliferative cells, a slight decrease is observed from 1 Gy onward, followed by a recovery at 5 Gy. This non-monotonic trend may reflect the interplay between radiation-induced transient growth arrest, which can reduce motility, and adaptive or stress-response mechanisms that partially restore migratory capacity at higher doses [38]. However, interpretation must remain cautious in the context of this study, as confirmation of this hypothesis would require additional experiments including doses exceeding 5 Gy. Based on the literature, the most strongly supported conclusion for MCF7 cells is the following: (i) a single dose of 2.3 Gy does not increase MCF7 cell migration (single-cell tracking and migration assays) [39]. (ii) Pro-migratory effects may arise indirectly through soluble factors or through a radiation-induced senescence-associated secretory phenotype at 6 Gy [40]. The most robust conclusion within this interval remains the absence of a detectable effect around 2 Gy and an increase around 6 Gy, as observed in the present study. It should also be noted that diffusion/migration measurements vary substantially depending on the cell line, the experimental protocol employed, the operational definition of diffusion, the post-irradiation time window considered, and the radiation doses applied, which collectively complicate direct comparisons across studies.

Unlike transiently arrested trees, where ITA and RPTA cells show markedly different behaviors, it was not necessary to differentiate between initial proliferative cells and their progeny. For simplification, they were grouped into a single proliferative category. The bioparameters were essentially identical, except that the initial cells at the beginning of the movie appeared smaller due to initial adhesion to the well plate. Moreover, comparing primary proliferative cells was challenging due to their low number relative to secondary proliferative cells; no biologically significant differences were detected.

## Conclusion

Time-lapse videomicroscopy, combined with automated lineage reconstruction using the CLT algorithm, provides a powerful approach to analyze the heterogeneous responses of cancer cells to irradiation, without using time-consuming invasing and costly bio-molecular methods. This methodology enables the identification and quantification of distinct subpopulations based on their behavior over time (proliferative, transiently arrested, and long-term arrested). The robustness of lineage reconstruction across experimental conditions confirms the reliability of the CLT algorithm and highlights its potential for large-scale single-cell analysis. This approach allows the investigation of intrinsic variability within clonal populations, revealing cell-to-cell differences that are masked in conventional population-based assays. By maintaining simple and controlled culture conditions, it provides a direct view of the dynamic processes underlying radiation-induced adaptation, without the need for additional molecular labeling.

Irradiation induces changes in MCF7 cells phenotypes, with increasing doses leading to larger cell areas, reduced diffusion, and a shift from proliferative to transiently arrested and long-term arrested phenotypes. Surface area and diffusion analyses reveal that long-term arrested cells exhibit maximal hypertrophy and minimal diffusion, transiently arrested cells show intermediate enlargement and partial diffusion recovery, with their daughter cells regaining a profile close to that of proliferative cells, which maintain smaller size and higher diffusion. Within each cell state, cell surface increased with higher doses, while diffusion did not show a clear dependence on dose. All observed differences between cell categories and across doses are statistically significant, highlighting the heterogeneous and category-specific cellular responses to irradiation. Such variability in individual responses may also underlie the emergence of radioresistant tumor subpopulations and, potentially, the proliferation of metastatic cells when these originate from particularly resilient cellular phenotypes.

This strategy can be readily extended to other experimental contexts and other cell lines, including the evaluation of radio-enhancing nanoparticles, combined treatments, or co-culture systems. The same framework can be adapted to explore intercellular communication effects, such as bystander signaling, or to assess radiation responses in mixed populations of normal and cancerous cells. Furthermore, it opens new opportunities to study FLASH irradiation and other emerging modalities at single-cell resolution.

## Supporting information

S1 Appendix CellLineageTrack algorithm and data availability. (PDF)

S2 Appendix CLT pipeline: Cell segmentation using Cellpose. (PDF)

S3 Appendix CLT pipeline: Cell tracking. (PDF)

S4 Appendix CLT pipeline: Cell linking. (PDF)

S5 Appendix Filtering and selection of relevant datasets. (PDF)

S6 Appendix Segmentation and tracking metrics; lineage tree reconstruction. (PDF)
