# Supplementary material for: Videomicroscopy reveals individual response of MCF7 cells to X-ray irradiation
Source: PLoS One. 2026 Apr 15;21(4):e0345480. doi: 10.1371/journal.pone.0345480 (PMC13082645; doi:10.1371/journal.pone.0345480)
Supplement: S1 Appendix — (PDF) [file pone.0345480.s001.pdf]

S1 Appendix. CellLineageTrack algorithm and data availability.

CellLineageTrack allows users to input their TIFF microscopy videos, select custom analysis parameters, and automatically generate a complete dataset of individual cellular measurements. At each stage of the workflow, verification videos are produced to facilitate quality control and ensure consistency with the original data. The software is open-source, licensed under the Apache License Version 2.0 and available at: `https://gitlab.in2p3.fr/josephine.courouble/celltrack`

For detailed installation instructions, usage guidelines, please refer to the README file provided in the repository.

The movies are publicly available at `https://mov.in2p3.fr/data.html`, where both raw microscopy movies and lineage tree reconstruction movies are provided in MP4 format. The CSV files used to generate the figures are also available. Original TIFF files are available upon request.
