# Supplementary material for: Videomicroscopy reveals individual response of MCF7 cells to X-ray irradiation
Source: PLoS One. 2026 Apr 15;21(4):e0345480. doi: 10.1371/journal.pone.0345480 (PMC13082645; doi:10.1371/journal.pone.0345480)
Supplement: S2 Appendix — (PDF) [file pone.0345480.s002.pdf]

S2 Appendix. CLT pipeline: cell segmentation using Cellpose.

Microscopy images were initially acquired in the proprietary `vsi` format and converted to the open `ome.tif` standard. To improve segmentation robustness, raw 16-bit TIFF images were systematically converted into 8-bit PNG images after automated contrast adjustment, including local background estimation, percentile-based intensity clipping, and signal-to-noise normalization. This preprocessing homogenizes image contrast across movies and experimental conditions, enhances cell boundaries, and significantly improves Cellpose segmentation performance compared to direct use of raw TIFF images.

Model training was performed using these pre-processed PNG images and the graphical interface of Cellpose. For each experimental condition, 3 out of the 36 available movies were randomly selected. Separate segmentation models were trained for different temporal windows (0-25 h, 25-50 h, 50-75 h, and beyond 75 h). Each model was trained using 12 annotated images from the initial 3 movies, at different times, corresponding to at least several hundred cells.

This temporal stratification was chosen to account for progressive changes in cell morphology throughout the experiment. While experimental conditions may also influence cell shape, variations in imaging conditions were found to be primarily driven by the acquisition time within the movie.

The generic Cellpose `cyto` model was first applied with an initial cell diameter set to 30 pixels. The resulting segmentation masks were then manually corrected to (i) separate merged cells, (ii) refine imprecise boundaries, and (iii) add missed cells. These corrected masks were used as ground-truth data for supervised training.

The training parameters were set as follows: learning rate of 0.1, weight decay of 10^{-4}, and 100 training epochs.

Cellpose version 2.2.3 was fixed for the entire study to ensure numerical stability and reproducibility of the results, particularly for computations performed on the IN2P3 computing center. Freezing the software version also guarantees strict comparability of segmentation performance and network outputs across datasets and throughout the development of the cell-tracking algorithm. The trained models are available on gitlab.
