# Supplementary material for: Videomicroscopy reveals individual response of MCF7 cells to X-ray irradiation
Source: PLoS One. 2026 Apr 15;21(4):e0345480. doi: 10.1371/journal.pone.0345480 (PMC13082645; doi:10.1371/journal.pone.0345480)
Supplement: S3 Appendix — (PDF) [file pone.0345480.s003.pdf]

S3 Appendix. CLT pipeline: cell tracking.

The tracking function pipeline (Fig 1) proceeds as follows:

- **Step 1: Barycenter Computation**
  Identify the segmentation masks corresponding to individual cells, and compute their barycenters.
- **Step 2: Nearest Neighbour Association**
  Identify nearest neighbour pairs and filter them based on a predefined distance threshold (`distance_max_in_micrometer`) to retain only plausible associations.
- **Step 3: Conflict Detection and Classification**
  Detect and classify matching conflicts:
  - **Case A**: Unique or absent matches.
  - **Case B**: Multiple associations.

  Cell associations are determined by minimizing a total cost function, equally weighted between:
  - The Euclidean distance between cell barycenters (Equation 1).
  - The morphological dissimilarity of cell contours (Equation 2).

  The combined association weight is defined in Equation 3.
  `generate_pairs_of_cell_id()` Solves an optimal matching problem by selecting cell pairings that minimize the total associated cost.

$$\Delta\rho_1 = \frac{\sqrt{\left(\bar{x}^{(t)} - \bar{x}^{(t-1)}\right)^2 + \left(\bar{y}^{(t)} - \bar{y}^{(t-1)}\right)^2}}{d_t} \tag{1}$$

  $\bar{x}^{(t)}, \bar{y}^{(t)}$: barycenter coordinates at time t
  d_t : distance threshold (`distance_max_in_micrometer`)

$$\Delta\rho_2 = \frac{\sqrt{\left(\sum_{i=1}^{N}(x_i^{(t)} - x_i^{(t-1)})\right)^2 + \left(\sum_{i=1}^{N}(y_i^{(t)} - y_i^{(t-1)})\right)^2}}{S} \tag{2}$$

  S: surface of cell at time t
  N: number of contour points
  $x_i^{(t)}, y_i^{(t)}$: contour coordinates at time t

$$w = \frac{\Delta\rho_1 + \Delta\rho_2}{2} \tag{3}$$

  $\Delta\rho_1$: centroid displacement between $t-1$ and t
  $\Delta\rho_2$: contour deformation between the cell at $t-1$ and t
  w: total association weight between the two cell instances

- **Step 4: Assignment of Unique Identifiers**
  Assign a unique identifier to each tracked cell. In this example, cell 1 was lost during tracking, cells 2 to 5 were successfully tracked, and cell 6 was identified as a newly appearing cell.

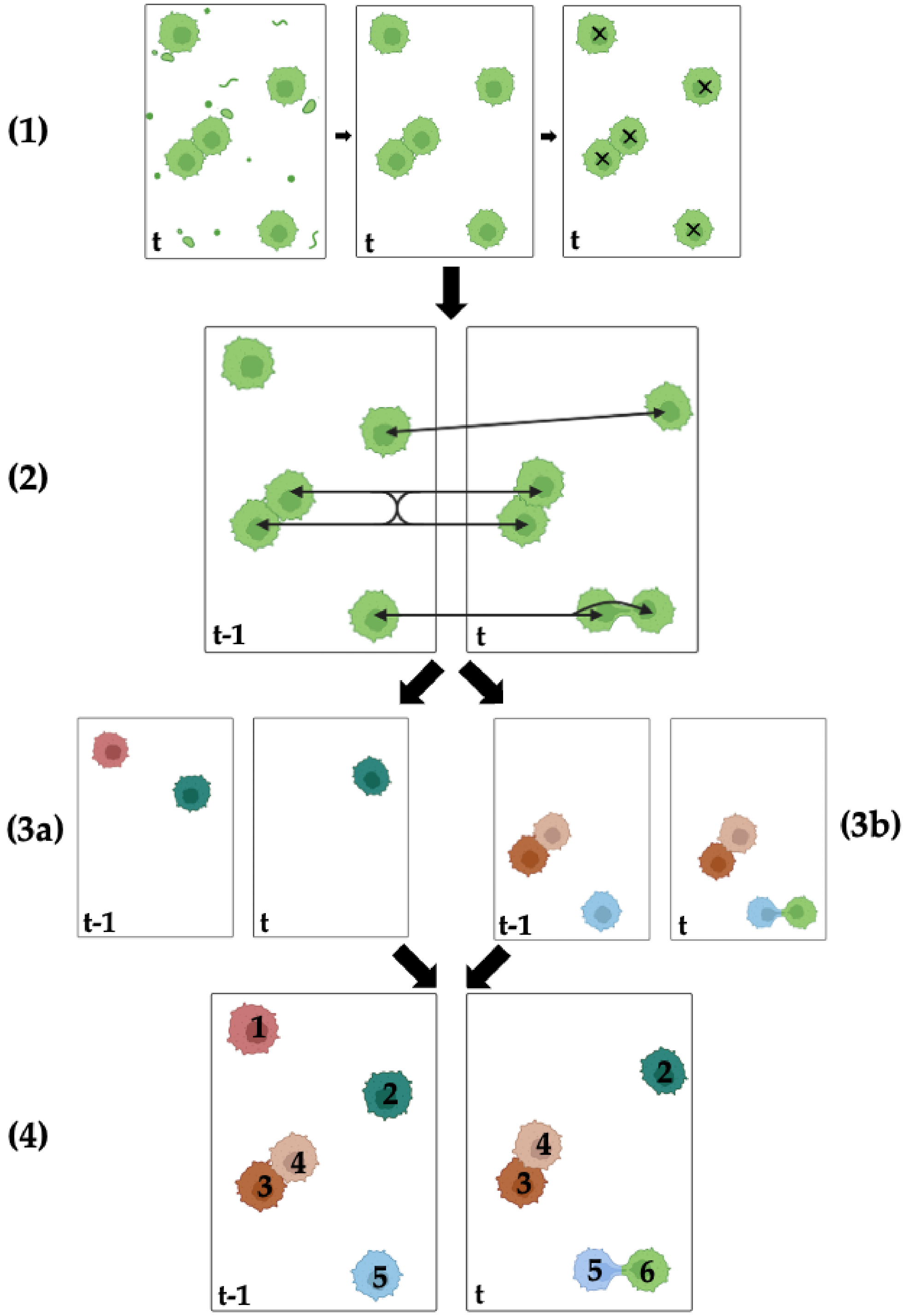

Fig 1. Overview of the tracking function workflow
