# Supplementary material for: Videomicroscopy reveals individual response of MCF7 cells to X-ray irradiation
Source: PLoS One. 2026 Apr 15;21(4):e0345480. doi: 10.1371/journal.pone.0345480 (PMC13082645; doi:10.1371/journal.pone.0345480)
Supplement: S4 Appendix — (PDF) [file pone.0345480.s004.pdf]

S4 Appendix. CLT pipeline: cell linking.

The `linking` function is structured into four main components:

- **Step 1: data harvesting**
  Based on the segmentation file refined by the tracking function, the algorithm constructs a dataframe that includes both global and time-resolved data for each cell. Global features include the frame of appearance, frame of disappearance, and total number of frames where the cell is present in the segmentation.
  Time-resolved features include presence at each time point t, centroid position (x, y), cell surface area, and fluorescence intensity.

- **Step 2: find cell origin**
  The goal of the second step (Fig. 1) is to identify the origin of any newly appearing cell (e.g., cell 1) at time t.

  - For $t = 0$, no assumption is made and no mother is assigned (Case 1).
  - For $t > 0$, a potential mother cell is searched; in some cases, no candidate is found (Case 2).
  - When a candidate is found, the biological plausibility of the association is assessed, considering that segmentation artifacts, such as over-segmentation, are a source of error. When the potential mother cell disappears just after the new cell appears (Case 3a), or when the newly appearing cell disappears immediately (Case 3b), this suggests that the segmentation is not accurate. In both of these subcases, the same cell (e.g., cell 0) is observed before and after the event, confirming the occurrence of over-segmentation, which is then corrected (Case 3).
  - Finally, when the post-appearance behavior is consistent, a division event of the potential mother cell is confirmed (e.g., cell 0) and assign distinct labels to the daughter cells (e.g., cells 1 and 2), differentiating them from the mother cell (Case 4).

- **Step 3: flags attribution**
  The third part assigns flags that describe appearance and disappearance events:

Table 1. Description of appearance, disappearance, and border-related flags used during cell linking.

**Category**	**Flag**	**Description**
Appearance	`app_init`	Initial appearance at $t = 0$
Appearance	`app_mitosis`	Appearance following a cell division
Appearance	`app_spont`	Spontaneous appearance without a defined origin
Disappearance	`dis_end`	Cell is still present at $t = t_{\max}$
Disappearance	`dis_mitosis`	Cell disappears after dividing
Disappearance	`dis_death`	Cell is lost during tracking
Border	`border`	Cell approached image border during its lifetime
Border	`app_border`	Appearance near the image border
Border	`dis_border`	Disappearance near the image border

Table notes: Flags are grouped by category (appearance, disappearance, border). Each flag indicates specific events or conditions used during cell linking.

Appearance and disappearance flags are mutually exclusive, whereas border flags are independent and may coexist with any other flag.

- **Step 4: tree identifier attribution**
  Finally, the fourth part of the function assigns a unique `tree` identifier to each group of cells that share a common mother cell, defining clonal lineages, using `doTree()`.

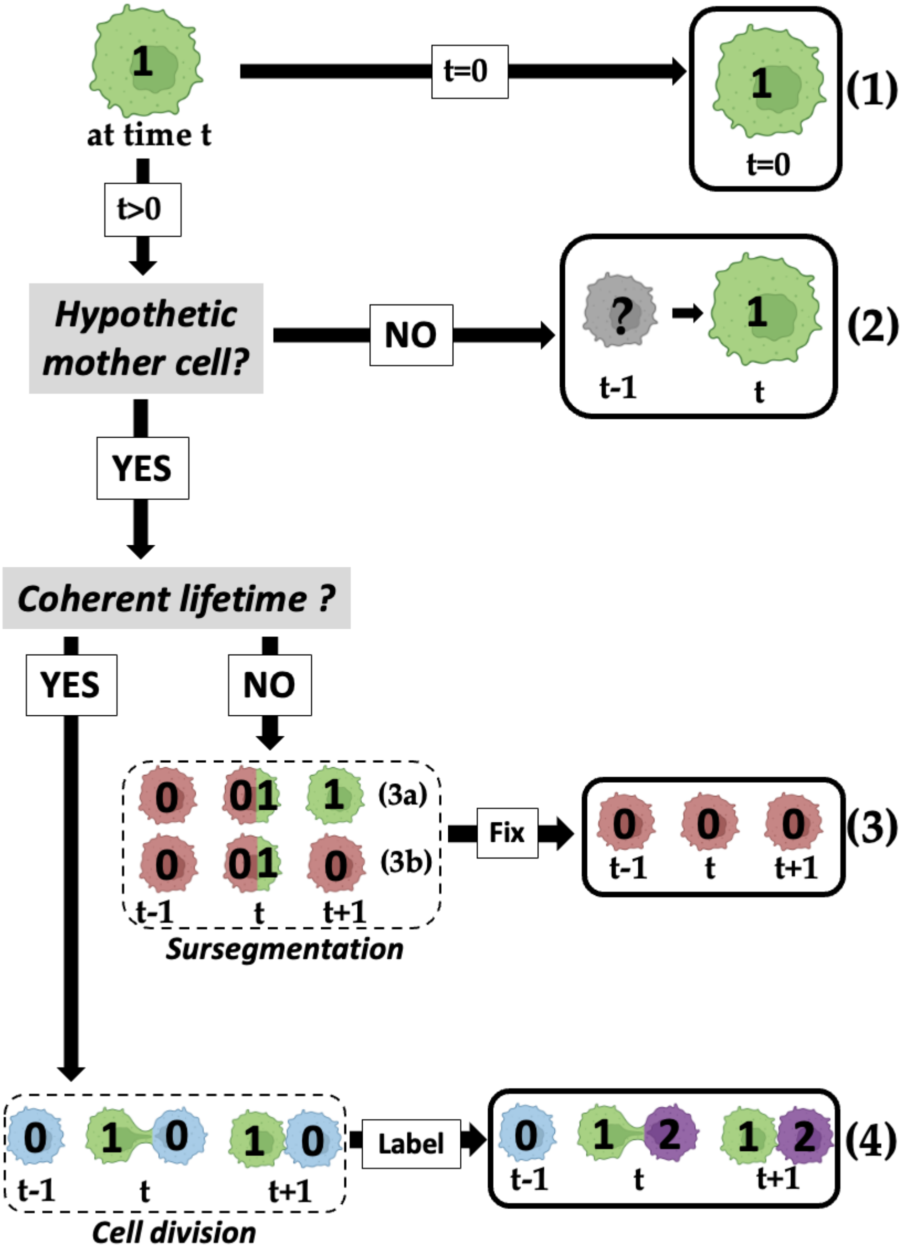

Fig 1. Overview of the step 2 linking function workflow
