# Supplementary material for: Videomicroscopy reveals individual response of MCF7 cells to X-ray irradiation
Source: PLoS One. 2026 Apr 15;21(4):e0345480. doi: 10.1371/journal.pone.0345480 (PMC13082645; doi:10.1371/journal.pone.0345480)
Supplement: S5 Appendix — (PDF) [file pone.0345480.s005.pdf]

S5 Appendix. Filtering and selection of relevant datasets.

The performance of the CLT algorithm was first evaluated through visual inspection of multiple time-lapse recordings to identify the main sources of errors. Three primary sources were observed: (i) overlapping cells, (ii) cells entering or leaving the field of view at the edges of the video, and (iii) rare biological events

(i) Cells tend to overlap particularly when their distribution is heterogeneous, leading to locally high confluence in specific regions, even if the overall confluence of the well remains low. This heterogeneity increases exponentially over time. To minimize the impact of locally high confluence, two complementary strategies were applied: first, only movies in which cells were initially distributed relatively uniformly were selected; second, the duration of the recordings was truncated once cells had proliferated sufficiently. The confluence criteria was based on the number of neighbours of cells at a given distance. Initially, 27 movies were acquired for control condition and 36 movies were acquired for irradiated conditions. Among these, the recordings that met the confluence criteria were as follows: for the 0 Gy condition, 27 movies were analyzed, truncated at 72 h; for 1 Gy, 33 movies were analyzed, truncated at 84 h. For higher doses, recordings could be analyzed up to 96 h, with 33 movies for 2 Gy, 34 movies for 3 Gy, 36 movies for 4 Gy, and 36 movies for 5 Gy.

(ii) Given the limited field of view, clusters of cells at the edges of the field accounted for approximately 20% of all cells over a few days. These cells and their lineage trees were excluded from the analysis.

(iii) To further ensure data quality, additional filtering steps are implemented for rare biological events, such as daughter cells remaining attached after division, tri-mitoses or cells moving unusually fast. All those events (approximately 5% of the dataset) occur infrequently at the biological scale and are not the primary focus in this study. First, lineage trees with at least a cell of unidentified origin were excluded. Second, lineage trees containing cell cycles shorter than 5h were excluded, as no biologically plausible cycles shorter than this duration were observed; such cases generally result from residual cell overlap or rare biological events described above.

After applying these criteria, approximately 75% of lineage trees remained, corresponding to datasets that were fully consistent with this visual observations. Although this filtering is conservative, it ensures that the analyzed sample is both reliable and biologically meaningful.
