# Supplementary material for: Videomicroscopy reveals individual response of MCF7 cells to X-ray irradiation
Source: PLoS One. 2026 Apr 15;21(4):e0345480. doi: 10.1371/journal.pone.0345480 (PMC13082645; doi:10.1371/journal.pone.0345480)
Supplement: S6 Appendix — (PDF) [file pone.0345480.s006.pdf]

S6 Appendix. Segmentation and tracking metrics; lineage tree reconstruction.

As show in Fig. 1, within the same irradiated population and the same cell cluster, some cells retain a proliferative phenotype whereas others exhibit enlarged morphologies and fail to divide. This strong heterogeneity represents a major challenge compared to classical tracking benchmarks, which typically involve homogeneous bacterial populations or short-term recordings of control cells [1–3].

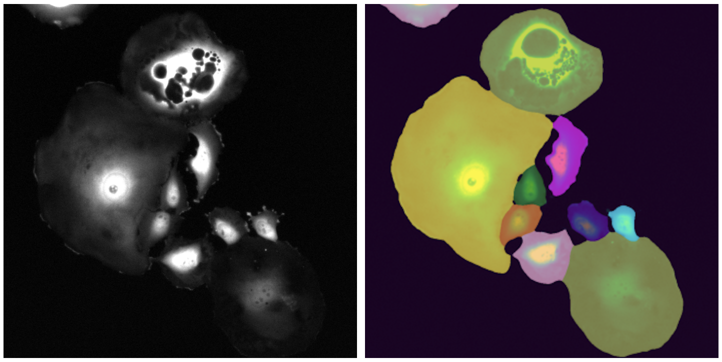

(a) **(b)**

Fig 1. Example of a cellular cluster at 5 Gy after 4 days. (a) Raw microscope image. (b) Corresponding segmentation computed with CLT.

For segmentation and tracking evaluation, the DetA and AssA metrics were computed [3]. While these metrics are widely used for detection and temporal association assessment, there is currently no consensus metric specifically designed to evaluate lineage tree reconstruction quality in heterogeneous mammalian systems. Although Cell-HOTA and related approaches have recently emerged [3], these were initially developed for bacteria dividing under controlled conditions. In contrast, irradiated mammalian cells may divide or arrest depending on dose, leading to lineage trees of highly variable topology.

Our primary objectives are therefore biological rather than purely algorithmic: (i) to verify that reconstructed cell cycle durations match the ground truth, and (ii) to ensure that each cell is assigned to the correct lineage tree. In our framework, lineage categorization depends exclusively on the initial ancestor cell defining each tree, and the analysis does not investigate intra-tree asymmetries; consequently, identity switches occurring between cells belonging to the same tree do not impact our downstream analyses. The experimental setting includes numerous atypical yet biologically relevant events which, taken together, represent a non-negligible fraction of the dataset, such as triple divisions or cases where cells detach while dividing, thereby complicating the unambiguous identification of mother-daughter relationships. In this context, the most critical requirement is to prevent mixing between cells originating from distinct lineage trees and to preserve accurate cell cycle duration, as this parameter directly underlies our tree categorization strategy.

Three complementary metrics are introduced, tailored to our study: (i) percentage accuracy of cell cycle duration reconstruction, (ii) percentage of correct lineage tree assignment (the most critical metric), (iii) percentage of correct mother-daughter detection events.

Although flexible, the CLT algorithm is not intended to be a general-purpose tracking framework but was specifically developed to address irradiation-induced heterogeneity in this experimental context.

Ground truth generation

A ground truth consists of manually validated segmentation masks and lineage annotations used as a reference for algorithmic evaluation. To ensure full control over annotation quality and reproducibility, a dedicated pipeline enabling the generation of custom ground truth datasets was developped. This pipeline is available on our GitLab repository.

Segmentation accuracy: DetA

Detection accuracy (DetA) is defined as [3]:

$$\mathrm{DetA}_\alpha = \frac{|\mathrm{TP}|}{|\mathrm{TP}| + |\mathrm{FP}| + |\mathrm{FN}|} \tag{1}$$

where α represents the minimum intersection-over-union (IoU) threshold, corresponding to the required percentage of shared pixels between predicted and ground truth masks.

- TP (True Positives): correctly detected objects with IoU $> \alpha$,
- FP (False Positives): predicted objects not corresponding to any ground truth object,
- FN (False Negatives): ground truth objects not detected.

The evolution of DetA as a function of α is shown in Fig. 2a. Importantly, DetA remains globally above 80% even for $\alpha = 0.95$, a threshold at which performance sharply degrades in the literature [4].

- For low α, DetA ranges between 90% and 100% across all irradiation doses. This does not imply that up to 10% of cells are excluded from the analysis; rather, it indicates that cells are temporarily misdetected during a small fraction of frames. These short detection gaps are corrected by the CLT framework through temporal interpolation, resulting in continuous trajectories suitable for downstream analysis.
- For high α, approaching 1 (corresponding to nearly perfect segmentation overlap), globally DetA remains above 80%, demonstrating that segmentation quality does not collapse as stricter matching criteria are imposed. Instead, DetA stabilizes at high α, indicating robust segmentation performance despite strong irradiation-induced phenotypic heterogeneity.

Temporal stability of segmentation at $\alpha = 0.8$ is shown in Fig. 2b. Notably, segmentation performance does not deteriorate over time and remains above 80% even after 4 days, across all irradiation doses, despite increasing morphological heterogeneity.

Tracking consistency: AssA

Association Accuracy (AssA) measures the temporal consistency of correctly tracked cells, evaluating whether predicted identities remain stable over time, is defined as [3]:

$$\mathrm{AssA}_\alpha = \frac{1}{|\mathrm{TP}|} \sum_{(g,p)\in \mathrm{TP}} \frac{|\mathrm{TPA}(g,p)|}{|\mathrm{TPA}(g,p)| + |\mathrm{FPA}(g,p)| + |\mathrm{FNA}(g,p)|} \tag{2}$$

where:

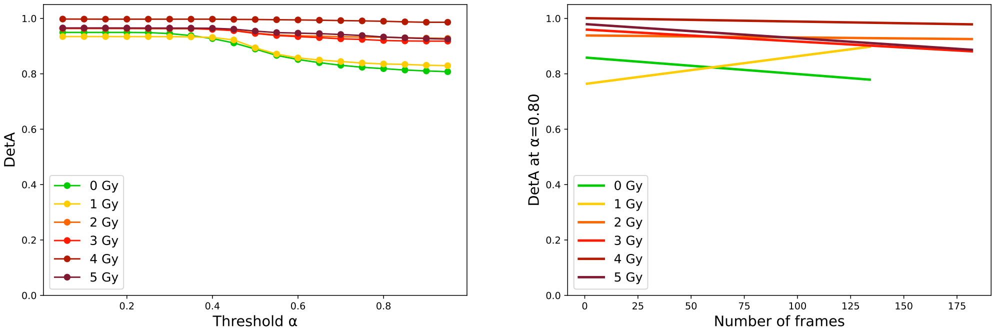

(a) **(b)**

Fig 2. Cell segmentation metric DetA, (a) evolution of DetA as a function of the IoU threshold α, (b) temporal evolution of DetA at $\alpha = 0.8$ over 4 days (with linear fit)

- TPA(g, p) (True Positive Associations): number of frames where predicted object p is correctly associated with ground truth object g,
- FPA(g, p) (False Positive Associations): frames corresponding to identity switches,
- FNA(g, p) (False Negative Associations): frames where g exists but is not associated with p (trajectory interruption).

For each matched pair (g, p), the fraction represents the proportion of time during which cell identity is correctly preserved. AssA is obtained by averaging this proportion across all matched objects.

As shown in Fig. 3, globally AssA remains above 80% for all irradiation doses, demonstrating robust and temporally coherent tracking despite long-term acquisition and irradiation-induced heterogeneity.

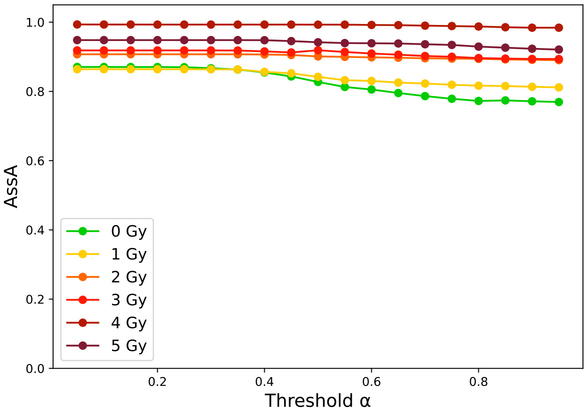

Fig 3. Association Accuracy (AssA) as a function of the IoU threshold α.

Lineage reconstruction accuracy (LinA)

To specifically evaluate lineage reconstruction quality, a dedicated analysis module was implemented. This module compares predicted lineage trees to the ground truth at three complementary levels: (i) correct lineage tree assignment, (ii) correct detection of division events, and (iii) accurate reconstruction of cell lifetime (cell cycle duration).

The percentage of cells assigned to the correct lineage tree as a function of irradiation dose is shown in Fig. 4(a). This metric evaluates whether cells originating from distinct ancestor cells remain properly separated, which is critical for our biological categorization framework. Across all irradiation doses, the percentage of correct lineage tree assignment remains above 95%. Importantly, the few observed discrepancies do not correspond to tree inversions (i.e., cells incorrectly assigned to a different existing lineage), but rather to cases where cells were manually linked to a lineage in the ground truth while the CLT algorithm classified them as spontaneous appearances and did not attach them to any tree. Because spontaneous appearances were excluded from the downstream biological analyses in this study, these rare cases do not impact our conclusions, and the effective percentage of correct lineage tree assignment can therefore be considered close to 100%. The percentage of correctly detected division events (mother-daughter relationships) is presented in Fig. 4(b), reflecting the algorithm's ability to identify mitotic events. Lineage assignment can be challenging because cells belonging to the same lineage tree may transiently detach synchronously just before division and subsequently reattach. Therefore, particular emphasis is placed on the percentage of correctly reconstructed lineage trees as a more robust metric. Finally, the percentage agreement in reconstructed cell lifetime relative to the ground truth is shown in Fig. 4(c). Accurate lifetime estimation is essential, as cell cycle duration directly underlies our lineage-based categorization strategy. Notably, the agreement in reconstructed cell cycle duration exceeds 80% for all irradiation doses (except for 5 Gy at 75%).

Together, these metrics provide a biologically meaningful evaluation of lineage reconstruction robustness across irradiation doses, complementing DetA and AssA by focusing on tree-level consistency rather than solely frame-wise detection and temporal association.

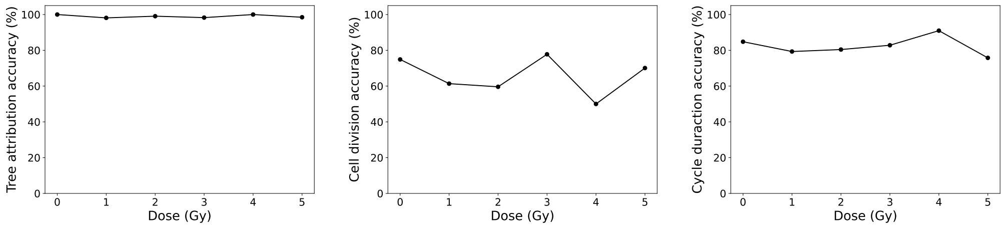

(a) (b) (c)

Fig 4. Lineage reconstruction accuracy (LinA) across irradiation doses. (a) Percentage of correct lineage tree assignment. (b) Percentage of correctly detected division events. (c) Percentage agreement in reconstructed cell lifetime.

References

1. Schwartz MS, Moen E, Miller G, Dougherty T, Borba E, Ding R, et al.. Caliban: Accurate cell tracking and lineage construction in live-cell imaging experiments with deep learning. Bioinformatics; 2019. Available from: `http://biorxiv.org/lookup/doi/10.1101/803205`. doi:10.1101/803205.

2. Li C, Xie SS, Wang J, Sharvia S, Chan KY. SC-Track: a robust cell-tracking algorithm for generating accurate single-cell lineages from diverse cell segmentations. Briefings in Bioinformatics. 2024 Mar;25(3):bbae192. Available from: `https:`

//academic.oup.com/bib/article/doi/10.1093/bib/bbae192/7659281. doi:10.1093/bib/bbae192.

3. O'Connor OM, Dunlop MJ. Cell-TRACTR: A transformer-based model for end-to-end segmentation and tracking of cells. Systems Biology; 2024. Available from: http://biorxiv.org/lookup/doi/10.1101/2024.07.11.603075. doi:10.1101/2024.07.11.603075.

4. Caicedo JC, Roth J, Goodman A, Becker T, Karhohs KW, Broisin M, et al. Evaluation of Deep Learning Strategies for Nucleus Segmentation in Fluorescence Images. Cytometry Part A. 2019 Sep;95(9):952-65. Available from: https://onlinelibrary.wiley.com/doi/10.1002/cyto.a.23863. doi:10.1002/cyto.a.23863.
